# Genome-wide association analysis of fiber-related traits and identification of diagnostic SNP markers differentiating spelt wheat (*Triticum spelta* L.) from bread wheat

**DOI:** 10.3389/fpls.2026.1907921

**Published:** 2026-08-07

**Authors:** Marianna Rakszegi, Viola Tóth, Sándor Tömösközi, Marietta Juhászné Szentmiklóssy, Edina Jaksics, Gyöngyvér Gell, Ágnes Bencze, Álmos Pance, Péter Mikó, Szabolcs Makai, Ildikó Karsai, András Cseh

**Affiliations:** 1 Agricultural Institute, Centre for Agricultural Research, Hungarian Research Network, Martonvásár, Hungary; 2 Research Group of Cereal Science and Food Quality, Department of Applied Biotechnology and Food Science, Faculty of Chemical Technology and Biotechnology, Budapest University of Technology and Economics, Budapest, Hungary

**Keywords:** cereals, diversity, fiber, GWAS, SNP array, spelt

## Abstract

**Introduction:**

Spelt wheat (*Triticum spelta* L.) represents a valuable genetic resource within hexaploid wheat due to its high genetic diversity and adaptation to low-input agricultural systems; however, the genetic basis of its dietary fiber composition remains poorly understood. This study aimed to identify genomic regions associated with key soluble fiber components, including water-extractable pentosans (arabinoxylans), β-glucan, and fructans, and to detect diagnostic SNP markers that may support targeted breeding for improved nutritional quality.

**Methods:**

A diverse panel of 255 genotypes, including spelt, bread wheat, and wild hexaploid accessions, was genotyped using a 25K SNP array and phenotyped over two consecutive years. Genome-wide association studies (GWAS) were performed with correction for population structure, and candidate genes were identified within ±1 Mb intervals surrounding significant SNPs based on functional annotations.

**Results:**

Significant phenotypic differences were observed between spelt and bread wheat, with spelt consistently exhibiting lower levels of soluble fiber components, whereas total pentosan content remained comparable between the groups. GWAS identified 24, 10, 17, and 20 significant marker-trait associations for total pentosan, water-extractable pentosan, β-glucan, and fructan content, respectively. Major genomic regions associated with fiber-related traits were detected on chromosomes 1B, 2A, 2B, 5A, 5B, 5D, 6B, and 7A. Several stable SNP markers exhibited consistent phenotypic effects across years and distinguished allele variants characteristic of spelt. Notably, one spelt-specific marker was associated with β-glucan content, highlighting potentially valuable allelic variation in spelt that could be exploited to improve grain quality. Candidate genes involved in carbohydrate metabolism and cell wall biosynthesis, including glycosyltransferases, cellulose synthase-like proteins, and fructan-related enzymes, were identified in the vicinity of associated loci. In addition, 16 diagnostic spelt-specific SNP markers enabled reliable discrimination between homogeneous spelt and bread wheat lines.

**Discussion:**

Overall, this study provides new insights into the genetic architecture of fiber-related quality traits in wheat and delivers molecular resources for marker-assisted breeding and the genetic identification of spelt germplasm.

## 1 Introduction

Spelt wheat (*Triticum spelta* L.) and common bread wheat (*Triticum aestivum* L.) are closely related allohexaploid species (AABBDD genome), yet they can be clearly distinguished at both morphological and genetic levels. One of the most fundamental differences lies in the allelic composition of the domestication-related Q locus on chromosome 5A. While bread wheat typically carries the brbrtgtgQQ genotype, spelt is characterized by the brbrtgtgqq combination, which results in its hulled grain, lax spike architecture, and reduced threshability (Tóth et al., 2022). These traits reflect a distinct evolutionary trajectory within the hexaploid wheat complex. In addition, spelt grains remain enclosed by glumes, conferring enhanced environmental resilience and suitability for low-input and organic farming systems (Takac et al., 2021).

Despite their shared genomic background, molecular studies have consistently demonstrated that spelt and bread wheat form distinct genetic pools. Early marker-based analyses using RFLP, SSR, and ISSR markers revealed both interspecific and intraspecific diversity, with particularly high variability observed within spelt populations (Siedler et al., 1994; Sun et al., 1998; Bertin et al., 2001; Golea et al., 2023). More recent high-throughput genotyping approaches, such as SNP array analyses, have confirmed the genetic differentiation between spelt and bread wheat, while also highlighting their close genomic relationship. Notably, spelt has been reported to harbor significantly higher sequence variation than bread wheat, particularly on chromosome 3B (Liu et al., 2018; Müller et al., 2018). This extensive genetic diversity, much of which exists within populations, underscores the value of spelt as a genetic reservoir for wheat improvement (Caballero et al., 2008; Longin and Würschum, 2014; Stefan et al., 2024).

Genetic studies in spelt have employed both biparental mapping populations and genome-wide association studies (GWAS). Classical linkage mapping using recombinant inbred line (RIL) populations derived from wheat × spelt crosses has identified numerous quantitative trait loci (QTLs) associated with agronomic and quality traits, including protein content, dough properties, kernel hardness, and pre-harvest sprouting resistance (Messmer et al., 1999; Zanetti et al., 2000, 2001). More recently, GWAS analyses of diverse spelt germplasm have identified genomic regions under selection for morphological traits and disease resistance, as well as loci associated with micronutrient accumulation (e.g. Zn and Fe) (Srinivasa et al., 2014; Würschum et al., 2017). Together, these studies indicate that, as in bread wheat, economically important traits in spelt are governed by complex polygenic architectures.

However, a critical gap in current knowledge lies in the genetic regulation of nutritional traits, particularly dietary fiber components, in spelt. In bread wheat, the genetic basis of major fiber fractions - such as arabinoxylan (AX), β-glucan, and fructans - has been extensively characterized, with numerous QTLs, candidate genes, and molecular markers identified (Ward et al., 2008; Shewry et al., 2011, 2013). For example, AX content has been linked to major QTLs on chromosomes 1B and 6B, as well as to genes belonging to glycosyltransferase families (GT43, GT47, GT61), which play key roles in cell wall biosynthesis (Mitchell et al., 2007; Lovegrove et al., 2013). In wheat, the primary QTLs for β-glucan biosynthesis are located on chromosomes 2A, 5B, and the group 7 homoeologs (7A, 7B, and 7D). The most critical genes associated with these regions belong to the CslF (Cellulose synthase-like) family, which are the main drivers of β-glucan accumulation in the grain. Similarly, fructan content has been associated with genomic regions such as chromosome 7A, harboring key biosynthetic genes including 1-SST (sucrose:sucrose 1-fructosyltransferase), 1-FFT (fructan:fructan 1-fructosyltransferase), and 6-SFT (sucrose:fructan 6-fructosyltransferase) (Huynh et al., 2012).

In contrast, equivalent studies in spelt are scarce, and the genetic determinants of its fiber composition remain largely unexplored. Although spelt exhibits considerable phenotypic variation in grain composition, including protein and carbohydrate fractions, the underlying molecular mechanisms - particularly for dietary fiber components - have not been systematically explored using GWAS or other high-resolution genomic approaches. As a result, breeding efforts targeting fiber-related traits in spelt still rely heavily on phenotypic selection and biochemical assays, which are time-consuming and less efficient.

Therefore, investigating genome-wide associations between dietary fiber components (such as AX, β-glucan, and fructans) and SNP markers in diverse spelt and bread wheat populations represents a novel and biologically relevant research direction. Given its high genetic diversity and distinct evolutionary background, spelt may harbor unique alleles and regulatory mechanisms that are absent or underrepresented in bread wheat. Identifying such spelt-specific variation will not only advance our understanding of fiber biosynthesis in cereals but also provide valuable tools for marker-assisted breeding aimed at improving nutritional quality.

In this study, we aimed to identify genomic regions associated with soluble fiber components in spelt and bread wheat, and to detect spelt-specific diagnostic SNP markers that enable reliable identification of spelt genetic background.

## 2 Materials and methods

### 2.1 Plant materials

A total of 255 genotypes -comprising 169 spelt wheat, 73 bread wheat, and 13 wild hexaploid accessions - were included in this study (**Supplementary Table 1**). These materials originated from 28 countries and exhibited substantial morphological diversity, particularly in spike characteristics such as the presence or absence of awns, spike color (white, red, grey, or black), and spike form, including *T. aestivum* (QQ allele type) and *T. spelta* (qq allele type). The wild hexaploid relatives were included as reference material to represent genetic variation within hexaploid wheat and to help assess genetic distances among hexaploid species. The wild relatives consisted of free-threshing subspecies of bread wheat: *T. compactum* (Host) Mackey (10 accessions), *T. macha* Dekapr. & Menabde (2 accessions), and *T. sphaerococcum* (Percival) Mackey (1 accession). The complete germplasm collection was phenotyped and analyzed in the first year of the study. Subsequently, a subset of 50 genetically diverse spelt and 50 bread wheat genotypes, together with seven wild hexaploid accessions showing close genetic similarity to bread wheat, was evaluated over two years. The second-year data were used to validate the marker-trait associations identified by the initial GWAS. Only SNP markers that consistently separated the population into significantly different phenotypic groups according to allele class in both years were retained for further analyses.

### 2.2 Analysis of the physical traits and milling

Thousand grain weight (TGW) was determined using a Marvin System instrument (MARViTECH GmbH, Wittenburg, Germany) in accordance with standard MSZ 6367/4-86 (MSZ 6367/4-86, 1986). Prior to milling, the samples were conditioned to a moisture content of 15.5%. Refined flour was produced using a Chopin CD1 Laboratory Mill (CHOPIN Technologies, Villeneuve-la-Garenne, France), while wholemeal flour was prepared with a Perten Laboratory Mill 3100 (PerkinElmer, Shelton, Connecticut, USA).

### 2.3 Dietary fiber measurements

β-glucan content of the wholemeal was determined enzymatically using spectrophotometric analysis with an Evolution 60S instrument (Thermo Fisher Scientific, Waltham, USA), following AACC Method 32-23.01 (American Association of Cereal Chemists, 1991b). Pentosans, including arabinoxylans in the flour, were quantified by a colorimetric method described by Douglas (1981). Fructan level of the white flour was measured using the Megazyme Fructan HK assay kit in accordance with AACC Method 32-32.01 (American Association of Cereal Chemists, 1995a). All analyses were performed in two technical replicates.

### 2.4 Genotypic characterization

Genomic DNA was extracted from 100 mg of fresh leaf tissue using the DNeasy^®^ Plant Mini Kit (Qiagen GmbH, Hilden, Germany), following the manufacturer’s protocol. The 25K Infinium genotyping analysis was carried out by TraitGenetics (SGS Institut Fresenius GmbH, Gatersleben, Germany). The selected 25K SNP set represents a subset of markers derived from the 90K SNP array and the 35K Axiom Wheat Genotyping Breeders’ Array (Wang et al., 2014; Allen et al., 2016).

### 2.5 Analysis of population diversity and association mapping

After filtering markers based on missing data (<10%), a total of 20,075 SNPs with known physical positions based on the IWGSC (International Wheat Genome Sequencing Consortium (IWGSC), 2014) RefSeq v1.0 assembly were retained for subsequent population structure analyses (PCA and kinship estimation) (Alaux et al., 2018). High-quality SNP data from 169 spelt, 73 bread wheat, and 13 wild hexaploid genotypes were combined into a single dataset to construct a genotype matrix. A kinship matrix was calculated using the VanRaden method as implemented in the Genome Association and Prediction Integrated Tool (GAPIT 3.0) in R (Wang and Zhang, 2021). Principal component analysis (PCA) was performed in GAPIT using default parameters, and the resulting principal components were used to assess population structure among the genotypes. A three-dimensional PCA plot was generated using the Plotly R package (Sievert, 2020), while SNP density was visualized using the CMplot R package (LiLin-Yin, 2018).

An additional minor allele frequency (MAF) filtering (MAF > 0.05) was applied prior to association analysis (GWAS), and genome-wide association mapping was performed using a compressed mixed linear model (cMLM) implemented in the GAPIT package (Lipka et al., 2012), based on a final dataset of 17,972 SNP markers. Population structure and cryptic relatedness were controlled by incorporating both principal components and a kinship matrix into the model to reduce false-positive associations (Kang et al., 2008). Model performance was evaluated using quantile-quantile (QQ) plots by comparing the expected and observed -log10(P) values. Statistical significance was determined using a threshold of −log10(P) ≥ 3.0 (P ≤ 0.001), this nominal significance threshold corresponds to an expected probability of approximately one false-positive association per 1,000 independent statistical tests (Zanke et al., 2015; Jung et al., 2021; Horváth et al., 2023).

Candidate genes were identified using a positional approach based on the IWGSC (International Wheat Genome Sequencing Consortium (IWGSC), 2014) RefSeq v1.0 wheat genome assembly (Appels et al., 2018). Candidate genes were searched within a 2 Mb genomic interval (1 Mb upstream and 1 Mb downstream) surrounding each significant SNP (−log10(P) ≥ 3.0) (Gahlaut et al., 2019; Gaur et al., 2022; Shahinnia et al., 2022). Gene models within these intervals were retrieved from the high-confidence annotation set, and functional information was assigned based on the IWGSC (International Wheat Genome Sequencing Consortium (IWGSC), 2014) RefSeq v1.0 annotation dataset. Trait-specific candidate genes were prioritized based on functional annotations related to carbohydrate metabolism and cell wall biosynthesis, including processes such as arabinoxylan, β-glucan, and fructan metabolism. Genes were ranked according to their physical proximity to associated SNPs, with the closest genes considered as primary candidates.

The expression profiles of candidate genes associated with WE-pentosan, β-glucan and fructan content were determined using publicly available data at wheat-expression.com (Ramirez-Gonzalez et al., 2018).

### 2.6 Statistical analysis

One-way and two-way ANOVA were performed to calculate the least significant difference (LSD) values. Specifically, a one-way ANOVA was used to determine the LSD for the phenotypic data of the full sample set. Phenotypic differences were compared between the species and among the different allelic variants. In contrast, a two-way ANOVA was applied to evaluate the genotype-by-year (G×E) interactions within the two-year phenotypic data. ANOVA as well as Hierarchical Cluster Analysis (using Ward’s method and Euclidean distance), were conducted with SPSS Statistics 27.0 (SPSS Inc., Chicago, IL, USA). Prior to multivariate analyses, the data were standardized using Statistica 6.0. Correlation analyses were performed, and box plots were generated using Microsoft Excel 2013 (Microsoft Corporation, Redmond, WA, USA).

## 3 Results

### 3.1 Variation in fiber content of spelt and common wheat

Variation of phenotypic data such as total- and water-soluble fibers (WE pentosan, β-glucan, fructan) was measured in a large set of spelt (*Triticum aestivum* ssp. *spelt*, n=169) and bread wheat (*Triticum aestivum*, n=86). The mean values of all water-soluble fibers (WE pentosan, b-glucan, fructan) differed significantly between spelt and wheat (LSD_5%_= 0.38, 0.21, 0.06, respectively), however the total pentosan content was similar in the two species (**Table 1**). Bread wheat exhibited higher levels of water-soluble fiber (WE pentosan, b-glucan, fructan) despite its significantly greater thousand-kernel weight compared to spelt, resulting in a smaller specific surface area. This means a 6.85 mg/g (0.685%) WE pentosan content, 5.39 mg/g (0.539%) β-glucan and 0.93% fructan content in average in spelt (**Table 1**), while its total pentosan content was 13.59 mg/g in the flour. The thousand kernel weight affected the b-glucan and fructan content in bread wheat (r_5%_=0.2476 and 0.2664, n=86), but had no effect in spelt (r_5%_=-0.0318 and 0.0241, n=169).

**Table 1:** Mean, standard deviation, minimum and maximum values of thousand kernel weight and fiber components (total (TOT) and water extractable (WE) pentosan, β-glucan, fructan) in spelt (n=169) and bread wheat (n=86) genotypes harvested in 2021.

	Spelt wheat n=169			Bread wheat n=86			All genotypes n=255		LSD
Descriptives	min-max	Mean	SD	min-max	Mean	SD	Mean	SD	spelt-wheat
TGW (g/1000 kernel)	27.41-54.71	39.19	4.91	29.01-52.62	42.00	5.59	40.13	5.30	1.36***
Total pentosan (mg/g)	10.22-20.63	13.59	1.80	9.47-20.85	13.33	1.94	13.50	1.85	0.55 ^n.s.^
WE pentosan (mg/g)	4.89-11.45	6.85	1.12	4.48-13.61	7.54	1.50	7.08	1.30	0.38**
β glucan content (mg/g)	4.00-7.43	5.39	0.62	4.77-10.98	6.58	1.01	5.79	0.95	0.21***
Fructan content (%)	0.29-1.51	0.93	0.20	0.40-1.52	0.98	0.25	0.95	0.22	0.06*

SD- standard deviation, LSD- Least Significant Difference.

significantly different at ***—p < 0.001, **—p < 0.01, *—p < 0.05 probability levels, n.s.—not significant.

**Figure 1:**
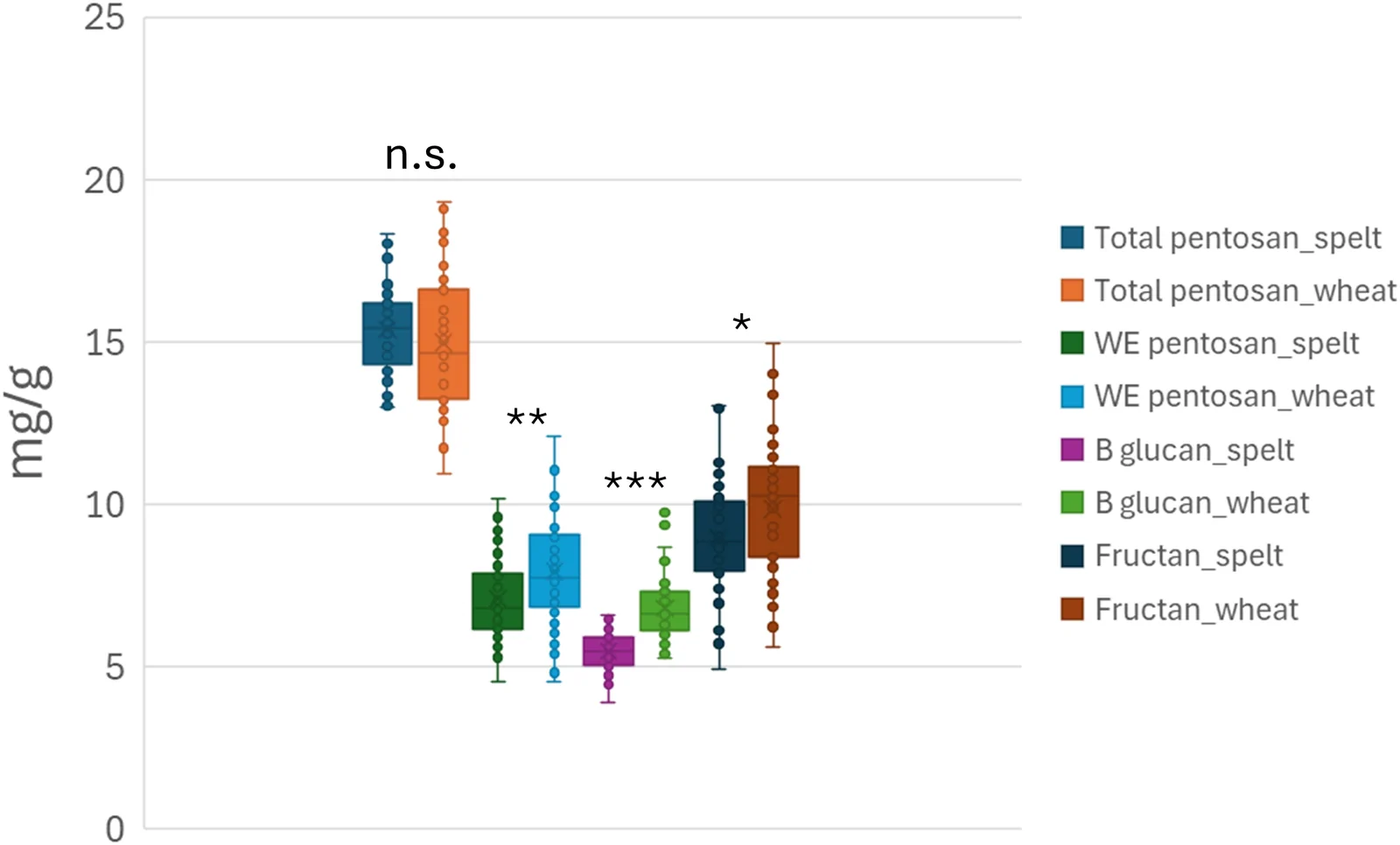
Box plots showing the amounts of fiber components (total pentosan, WE-pentosan, β-glucan, and fructan) in 50 spelt and 50 bread wheat genotypes, presented as mean values across two years (2021 and 2022). significantly different at ***—p < 0.001, **—p < 0.01, *—p < 0.05 probability levels, n.s.—not significant.

Two years data was also evaluated using fifty bread wheat and fifty spelt wheat genotypes (**Supplementary Table 2**). There was again a significantly lower amount of soluble fiber (WE pentosan, β-glucan, fructan) in spelt compared to wheat, while the amount of the total pentosan and the thousand kernel weight was similar in both species based on two-years average data and that was also the case in 2022. In 2021, only the β-glucan content showed significant difference between the two species and there was a small difference in their thousand kernel weight as well. The year affected the WE pentosan content in both spelt and wheat (LSD_5%_= 0.69, 0.54), and also affected the fructan content of spelt (LSD_5%_= 0.08) (**Supplementary Table 2**, **Figure 1**).

There was 7.10 mg/g (0.710%) WE pentosan content, 5.48 mg/g (0.548%) β-glucan and 0.90% fructan content in spelt in two-years average (**Supplementary Table 2**), while its total pentosan content was 15.38 mg/g in the flour.

Detailed quality analysis of these genotypes were summarized in Rakszegi et al. (2025).

### 3.2 Population structure and identification of spelt-specific SNP markers

**Figure 2:**
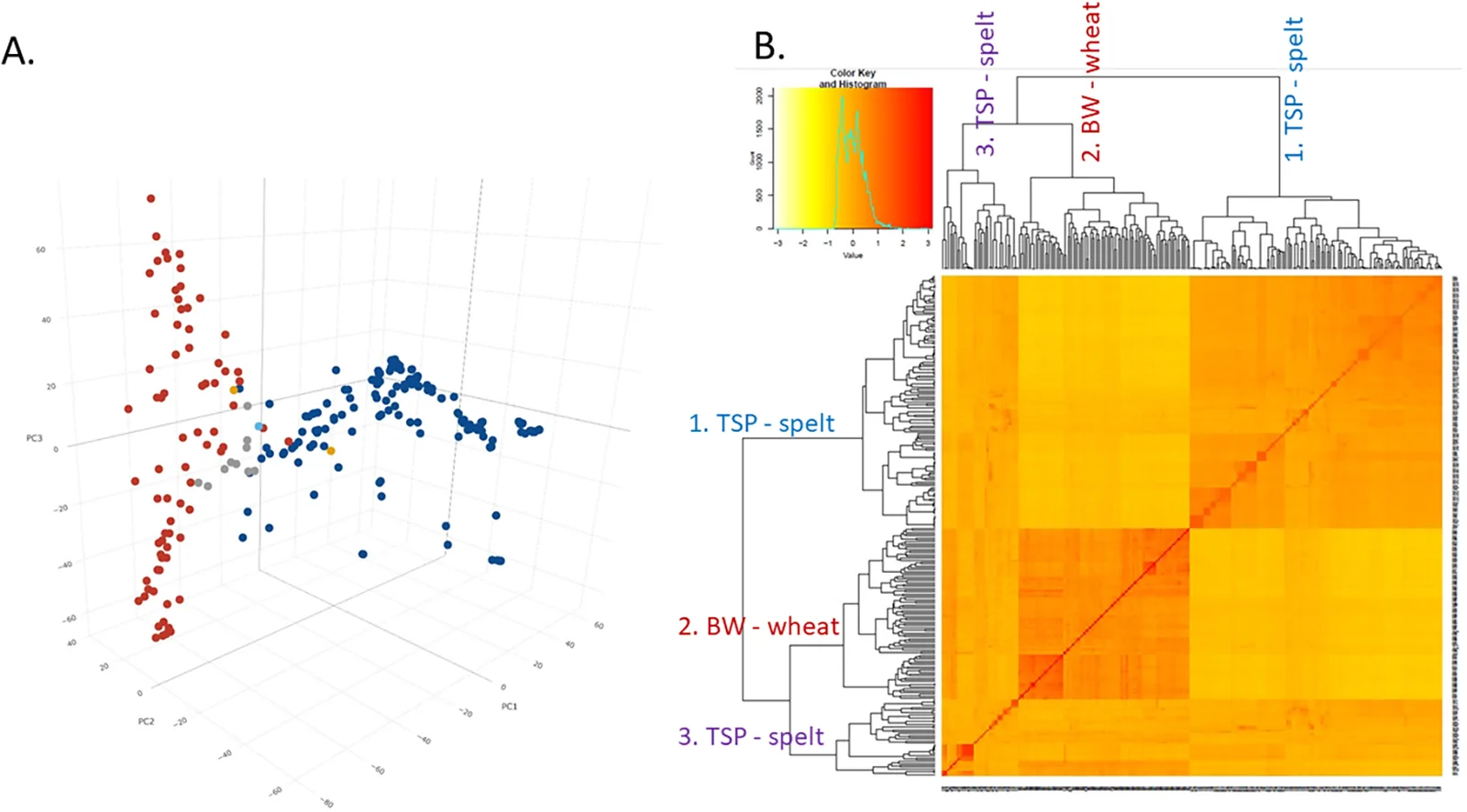
Population structure of the GWAS panel comprising 268 genotypes based on 25K SNP array analysis. **(A)** Principal component analysis (PCA) showing the genetic structure of bread wheat and spelt accessions. Bread wheat genotypes (n = 80) are indicated in red, spelt genotypes (n = 175) in blue, and wild hexaploid genotypes as follows: *T. compactum* (n = 10) in grey, *T. macha* (n = 2) in yellow, and *T. sphaerococcum* (n = 1) in light blue. **(B)** Heat map of the VanRaden kinship matrix. BW, bread wheat; TSP, *Triticum spelta*; TSP2, a distinct subgroup of spelt genetically closer to bread wheat.

The population structure of the GWAS panel, comprising 175 spelt, 80 bread wheat, and 13 wild hexaploid genotypes (n=268), was characterized using high-density genotyping data generated from a 25K SNP array. Principal component analysis (PCA) together with a kinship matrix calculated using the VanRaden method was applied to assess the genetic structure of the population based on 20,075 genome-wide SNP markers. PCA revealed a clear separation between spelt and bread wheat genotypes along the first principal component (PC1; **Figure 2a**), indicating strong genetic differentiation between the two groups despite their shared hexaploid genome background. Spelt genotypes formed a distinct cluster, clearly separated from bread wheat, which occupied an opposing region of the ordination space. Wild hexaploid accessions were positioned between the two groups but showed a closer affinity to bread wheat. The first three principal components explained 20.40%, 4.93%, and 4.19% of the total genetic variation, respectively, accounting for 29.52% overall (**Supplementary Figure 1**). Notably, bread wheat genotypes exhibited greater dispersion across principal components, particularly along PC2 and PC3, indicating higher intra-group genetic variability compared to spelt. In contrast, spelt formed a more compact cluster, suggesting a comparatively narrower genetic base. This pattern is consistent with the distinct domestication history and breeding trajectories of the two wheat types.

The VanRaden kinship matrix constructed for the 268 genotypes (**Figure 2b**) further supported the separation of spelt and bread wheat into two major groups (BW and TSP), while also capturing substantial variation at the genotype level. Notably, 40 accessions that had originally been classified as spelt based on gene bank records formed a distinct subgroup (TSP2-spelt) positioned closer to the bread wheat cluster in the kinship analysis. The genetic proximity of these accessions to modern bread wheat suggests possible historical introgressions or earlier hybridization events. Within the main spelt and bread wheat groups, 12 poorly clustered genotypes were identified, likely reflecting gene bank maintenance or classification errors. These accessions were therefore excluded from subsequent phenotypic analyses due to suspected misclassification. In addition, one waxy genotype was removed because it introduced substantial bias into the statistical evaluation. Consequently, a total of 255 genotypes were retained for further analyses.

### 3.3 Identification of spelt-specific markers

**Figure 3:**
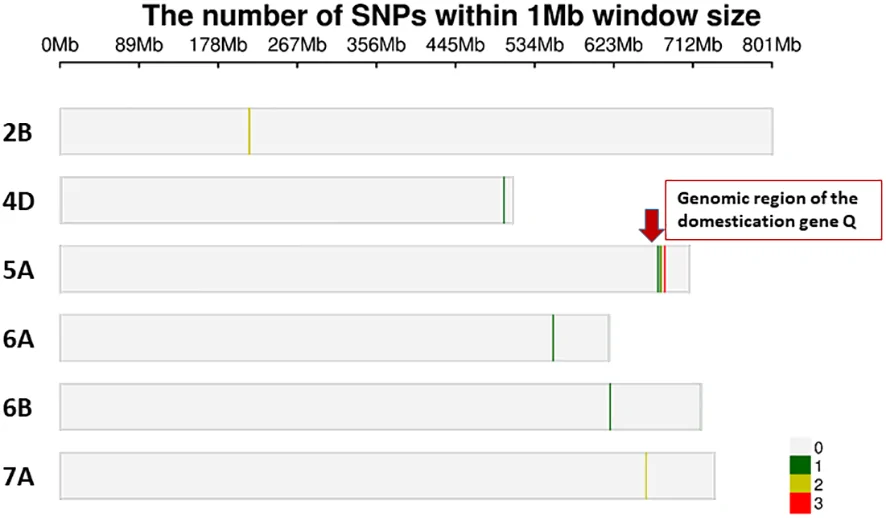
Chromosome-wise SNP density plot showing the distribution of 16 *T. spelta*-specific diagnostic SNP markers using a 1 Mb sliding window. The horizontal axis represents chromosome length (Mb), while the color scale indicates SNP density.

According to the clustering pattern of the kinship matrix, the TSP group was used to identify spelt-specific SNP markers. Within the TSP group, 389 SNP markers were identified that were conserved across all accessions, carrying the same allele in each genotype (**Supplementary Table 3**). None of these markers displayed an alternative allele consistently across all lines of the BW group compared with the spelt accessions. Therefore, we identified markers for which the spelt-associated allele was present in fewer than nine BW lines. This filtering approach resulted in the identification of 16 diagnostic SNP markers, the combined use of which enables the highly reliable identification of spelt accessions (**Figure 3**, **Supplementary Table 3**). Among the 73 BW lines, 31 carried at least one spelt-specific allele; however, only seven lines carried more than four simultaneously. The cultivar combining the highest number of spelt-specific alleles, GK-Bekes (Hungary), carried only eight of these alleles (**Supplementary Table 3**). The highest number of diagnostic markers was identified on chromosome 5A, where seven markers clustered within a genomic interval located approximately 22–30 Mb distal to the domestication-related Q locus. This suggests that the differentiation between bread wheat and spelt is associated with a broader haplotype structure within the 5A domestication region (**Figure 3**). Although 105 SNP markers were located closer to the Q locus on the 25K SNP array, none fulfilled our diagnostic marker criteria. This indicates that a distal haplotype block provides substantially greater discriminatory power between bread wheat and spelt than SNPs immediately flanking the *Q* gene.

### 3.4 Analysis of marker trait association

**Figure 4:**
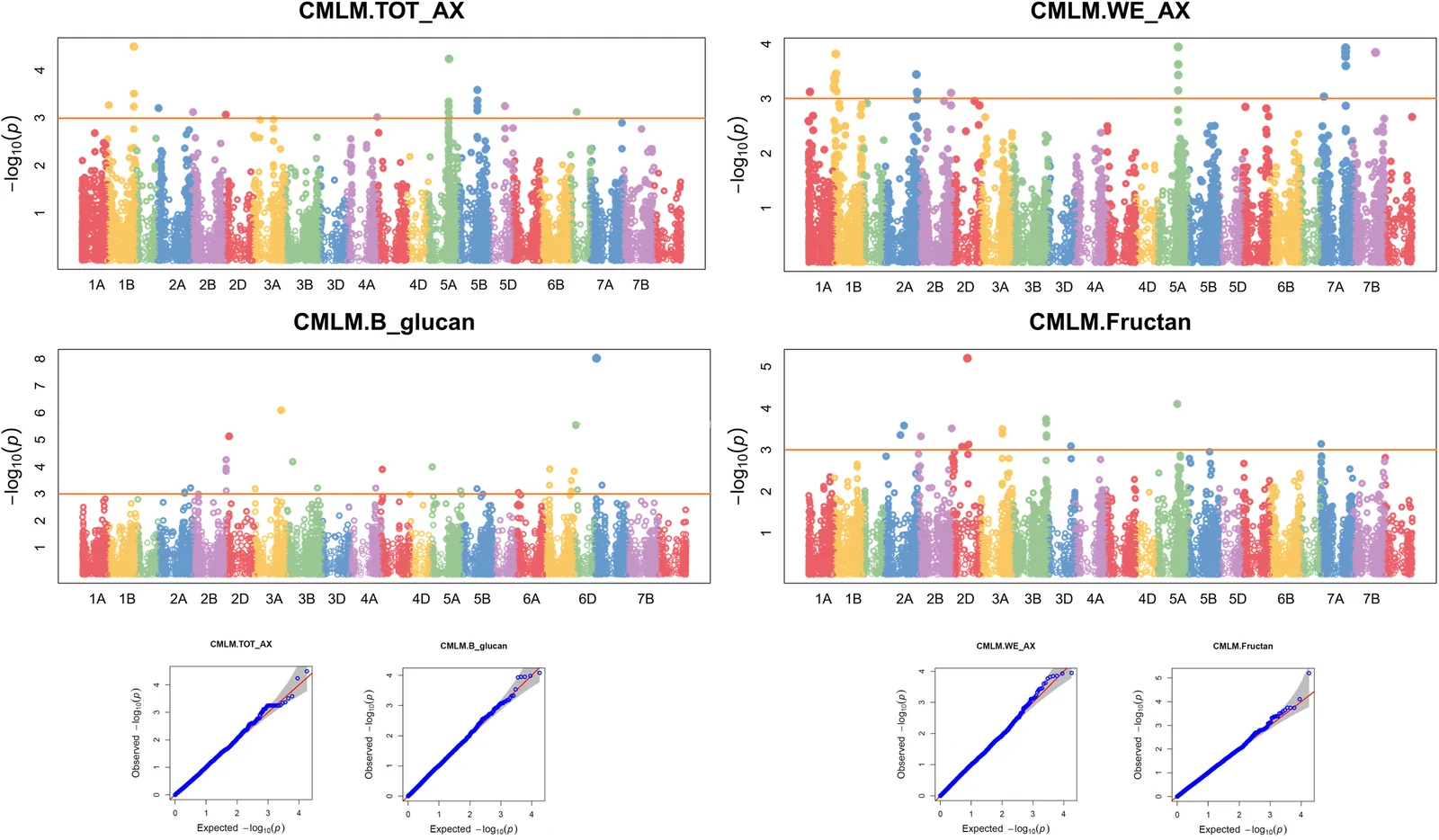
Manhattan and corresponding Q–Q plots for fiber-related traits in wheat, including total pentosan, water-extractable pentosan (arabinoxylan), β-glucan, and fructan content.

Association analysis of total and soluble fiber components with SNP markers was performed using the compressed Mixed Linear Model (cMLM) implemented in the GAPIT package in R. A total of 24 marker-trait associations (MTAs), representing seven genomic regions associated with total pentosan content, were identified (**Supplementary Table 4**, **Figure 4**). Marker-trait associations were considered significant when both the −log10(p) value and the percentage of phenotypic variation explained (PVE%) exceeded 3. The associated markers were located on chromosomes 1B, 2A, 2B, 2D, 5A, 5B, and 5D. The highest number of MTAs was detected on chromosome 5A (13), followed by chromosomes 5B (4) and 1B (3). Although a relatively high number of significant MTAs was identified, no significant differences in total pentosan content were observed between the two allelic variants in either individual year (2021 and 2022) or in the two-year mean values. Therefore, only markers showing significant differences consistently across all three datasets were highlighted in this study (**Supplementary Table 4**).

Water soluble fibers showed more significant associations in both years than the total amount. Ten MTAs were identified for WE pentosan, 17 for β-glucan and 20 for fructan representing 5, 9 and 9 regions associated with these traits, respectively (**Supplementary Table 4**).

The marker regions of WE pentosan were located on chromosomes 1B, 2A, 2B, 7A, and 7B, from which the highest number of MTAs was found on chromosome 7A (4) and 2A (3).

The marker regions of β-glucan were located on chromosomes 2A, 2B, 4B, 5A, 5B, 6B and 7A, from which the highest number of MTAs was found on chromosome 2A, 2B and 6B (4-4), having two regions on 2B and 6B.

The marker regions of fructan were located on chromosomes 2A, 2B, 2D, 3A, 3B, 3D, 5A and 7A, from which the highest number of MTAs was found on chromosome 3B (6) followed by 2D (4) and 3A (4), having two markers on 2A (**Supplementary Table 4**).

From these MTAs, 6 validated markers showed significant effect across both years for water-extractable pentosan and β-glucan content, while eleven stable markers were identified for fructan content (**Table 2**, **Figure 4**, **Supplementary Figure 2**). However, no stable, significant allelic effects were detected for total pentosan content.

**Table 2:** Marker trait associations for dietary fiber components of the grain in GWAS panel (2021, 2022).

Trait association	Significant SNP markers	Chromosome	SNP marker position	.-log10 (p)	PVE%	Typical allele variant of spelt	Typical allele variant of wheat	Avg phenotype for spelt allele	Avg phenotype for wheat allele	LSD_5%_ _***_
WE pentosan	BS00087787_51	1B	50778575	3.46	3.07	C	T	7.82	9.65	0.2446
WE pentosan	AX-108804304	2A	735008728	3.44	3.13	G	A	7.51	8.61	0.2385
WE pentosan	IAAV942	7A	570182645	3.77	3.981	A	G	7.92	9.01	0.2395
WE pentosan	AX-108918650	7A	572236045	3.85	4.02	G	A	7.92	8.14	0.2390
WE pentosan	GENE-4898_208	7A	572869037	3.93	4.01	G	A	7.69	9.02	0.2390
WE pentosan	AX-95148416	7B	532663736	3.84	3.97	T	C	7.69	9.01	0.2395
b-glucan	BS00067878_51	2B	784621059	6.17	7.09	G	A	5.53	6.79	0.1472
b-glucan	Excalibur_c48404_59	2B	789868993	3.18	3.05	C	T	5.68	7.22	0.1559
b-glucan	wsnp_Ex_c15646_23969140	2B	789869145	3.18	3.05	A	G	5.68	7.22	0.1559
b-glucan	TG0010b	4B	30861559	3.95	5.35	C	T	5.65	7.53	0.1479
b-glucan	BobWhite_c33227_82	6B	640908043	3.03	4.49	C	T	5.44	5.62	0.1554
b-glucan	wsnp_Ex_c1276_2445537	6B	642348364	11.45	5.28	T	C	5.61	6.73	0.1536
fructan	AX-158536950	2B	776212162	3.52	5.54	C	A	0.93	1.10	0.03737
fructan	AX-158522714	2D	214051332	3.08	4.89	G	T	0.92	1.04	0.03729
fructan	TA003872-0714	2D	293142212	3.08	4.89	A	G	0.92	1.04	0.03729
fructan	AX-89640158	2D	340512100	5.20	13.25	G	A	0.91	1.03	0.03701
fructan	AX-108781921	2D	369482459	3.13	7.02	T	C	0.92	1.03	0.03735
fructan	BobWhite_c26893_161	3A	502268586	3.39	3.97	C	T	0.93	1.18	0.03663
fructan	wsnp_Ku_c3343_6206887	3A	502385093	3.39	3.97	A	G	0.93	1.18	0.03663
fructan	wsnp_Ex_c2678_4968284	3A	502386566	3.39	3.97	T	G	0.93	1.20	0.03663
fructan	wsnp_Ku_c3956_7237707	3A	502402793	3.51	5.48	C	T	0.93	1.13	0.03675
fructan	AX-158580215	3D	520823245	3.09	4.29	A	G	0.93	1.15	0.03663
fructan	AX-158567449	7A	4256169	3.14	3.79	T	C	0.92	1.08	0.03712

PVE%- percent phenotypic variation explained *** significant at 0.001 probability level.

The six most significant SNP markers associated with WE pentosan content were located on chromosomes 1B (BS00087787_51), 2A (AX-108804304), and 7B (AX-95148416), while three additional markers formed a closely linked cluster on chromosome 7A (IAAV942, AX-108918650, GENE-4898_208). These markers clearly distinguished between the two allelic variant groups, and the allele associated with lower WE pentosan content was characteristic of spelt genotypes (**Figure 4**, **Supplementary Figure 2**, **Table 2**).

The six most significant SNP markers associated with β-glucan content were located on chromosomes 2B (BS00067878_51, Excalibur_c48404_59, wsnp_Ex_c15646_23969140), 4B (TG0010b), and 6B (BobWhite_c33227_82, wsnp_Ex_c1276_2445537). Markers located on the same chromosome formed closely linked genomic regions, and the TG0010b SNP marker was located within the Rht1 gene. These markers clearly distinguished between the two allelic variant groups, and the allele associated with lower β-glucan content was characteristic of spelt genotypes (**Figure 4**, **Supplementary Figure 2**, **Table 2**).

The eleven most significant SNP markers associated with fructan content were located on chromosomes 2B (AX-158536950), 2D (AX-158522714, TA003872-0714, AX-89640158, AX-108781921), 3A (BobWhite_c26893_161, wsnp_Ku_c3343_6206887, wsnp_Ex_c2678_4968284, wsnp_Ku_c3956_7237707), 3D (AX-158580215) and 7A (AX-158567449). being different marker locations on 2D. These eleven markers clearly distinguished between the two allele variants, with the allele associated with lower fructan content being characteristic of spelt (**Figure 4**, **Supplementary Figure 2**, **Table 2**).

The eleven most significant SNP markers associated with fructan content were located on chromosomes 2B (AX-158536950), 2D (AX-158522714, TA003872-0714, AX-89640158, and AX-108781921), 3A (BobWhite_c26893_161, wsnp_Ku_c3343_6206887, wsnp_Ex_c2678_4968284, and wsnp_Ku_c3956_7237707), 3D (AX-158580215), and 7A (AX-158567449). The strongest association was detected for AX-89640158 on chromosome 2D, explaining 13.25% of the phenotypic variation. Allelic comparisons revealed that the marker variants associated with reduced fructan content were predominantly enriched in spelt genotypes (**Figure 4**, **Supplementary Figure 2**, **Table 2**).

**Figure 5:**
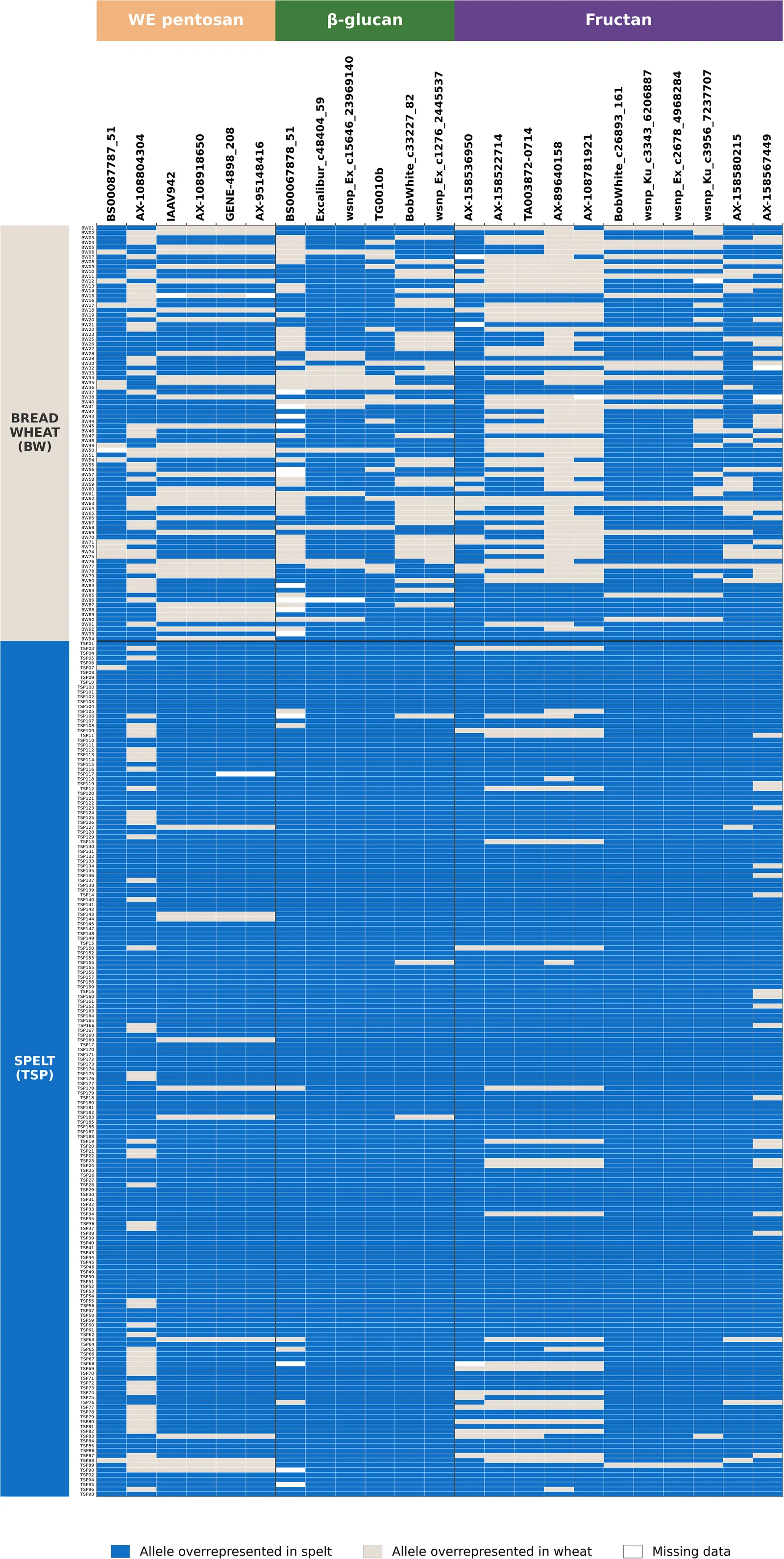
Allele combinations across genotypes for the identification of their association with high-fiber and low-fructan phenotypes. Blue cells highlight alleles overrepresented in spelt.

**Figure 5** shows the distribution of favorable, wheat-specific haplotype alleles across the analyzed genotypes and includes only markers with a phenotypic variation explained (PVE) value exceeding 3%. There was little difference among the PVE values of the six markers significantly associated with WE-pentosan content, which ranged between 3 and 4%. As no major-effect marker was identified, the individual and additive effects of the alleles do not reveal a clear pattern. Nevertheless, the genotypes with the highest WE-pentosan contents (13.61, 13.28, 12.11, and 11.08 mg/g in BW1, BW6, BW77, and TSP88 [‘Yumai-34’, ‘Mv-Marsall’, ‘Mv-Suba’, and ‘Vulpinum Alef.’], respectively) either carried at least four favorable alleles (IAAV942, AX-108918650, GENE-4898_208, and AX-95148416) or possessed the favorable allele of AX-108804304. This latter group included genotypes BW19, BW21, BW71, TSP66, and TSP106 (‘Cubus’, ‘Seu-Seun-27’, ‘Lynx’, ‘MVGB353’, and ‘Spelt-Fra-Oland’), with WE-pentosan contents of 11.20, 11.02, 11.21, 11.20, and 11.45 mg/g, respectively (Rakszegi et al., 2025). Genotypes with the lowest WE-pentosan content do not contain any favorable allele of the six markers (for example genotypes BW 26, 40, 43, 44, TSP 105, 118, 133, 148, 44, 45,46 or 98 (‘Hereward’, ‘Isengrain’, ‘Alabasskaja’, ‘Aurora’, ‘Brun’, ‘Roter-Grannenspelz’, ‘Tiroler-Roter-Dinkel-Dicht’, ‘Rottweiler-Dinkel-ST6’, ‘Oberkulmer/Schwabenkorn_1’, _2’ and_3’, ‘Rothweiler-Fruehkorn’) with values between 5.36-5.96 mg/g).

Six significant markers were identified for β-glucan content with PVE values exceeding 3%. Among them, BS00067878_51, TG0010b, and wsnp_Ex_c1276_2445537 exhibited the highest effects, explaining 7.09%, 5.35%, and 5.28% of the phenotypic variation, respectively. The genotypes with the highest β-glucan content contained at least the first two significant alleles (such as in BW7, 22, 35, 36, 40, 68, (‘NX02Y4530’, ‘Atay-85’, ‘Geronimo’, ‘Martonvásári-17’, ‘Isengrain’, ‘Flamura-85’) with β-glucan contents of 10.98, 9.6, 7.86, 10.15, 7.84 and 8.01mg/g), while the genotypes with the lowest β-glucan content did not contain any wheat-specific alleles (for example BW94, TSP10, 134, 15, 156, 22, 28, 38, (‘MVGB543’, ‘Ostar’, ‘Zeiners-Schlegeldinkel’, ‘Hercule’, ‘Bregenzer Roter Spelz’, ‘Franckenkorn’, ‘D-7-010-99-02’, ‘Stone’) with b-glucan contents of 4.27, 4.25, 4.28, 4.00, 4.19, 4.35, 4.01) (Rakszegi et al., 2025).

Eleven significant markers were identified in relation to fructan content of the seed with PVE value higher than three, from which AX-89640158, AX-108781921, AX-158536950 and wsnp_Ku_c3956_7237707 markers were the highest phenotypic effects (with 13.25, 7.02, 5.54 and 5.48 PVE values, respectively). In order to achieve a high fructan content, usually at least the presence of the two most significant markers are necessary. For example, samples BW 4, 5, 13, 22, 30, 56, 57, 63, 77 and TSP 12, 19, 65, 82, 87, 88 (‘Koreli’, ‘Lona’, ‘Thesee’, ‘Atay-85’, ‘Stephens’, ‘Renan’, ‘Glenlea’, ‘Vlasta’, ‘Mv-Suba’, ‘CH65384’, ‘BA7418’, ‘MVGB319’, ‘AlefeldII’, ‘Albo Velutinum’, ‘Vulpinum Alef. ‘) achieved fructan contents between 1.21 and 1.49%. The varieties with the lowest fructan content mostly do not contain any alleles for the 11 markers determinant in wheat and the fructan content of these samples ranges between 0.39 and 0.67% (for example BW 16, 83, 84, 86, 88, 92, TSP 8, 10, 25, 26, 27, 32, 33, 49, 51, 53, 64, 122, 123, (‘Ble-des-Domes’, ‘MVGB323’, 324’, 583’ and 322’, ‘Lyallpur’, ‘Lueg’, ‘Ostar’, ‘D-7-005-99-01’, -04’, -06’, ‘D-7-019-99-02’, ‘Cosmos’, ‘Oberkulmer/Sertel1’, ‘TSP04-09’, ‘TSP06-10’, ‘MVGB318’, ‘Brauner- Winter-Grannendinkel-Aus-Noerdlingen’, ‘Kipperhaus-Weisser-Spelz’) (Rakszegi et al., 2025).

### 3.5 Candidate gene identification

To identify putative functional candidates underlying fiber-related traits, all significant SNP markers were screened for annotated coding genes within a ±1 Mb interval using the IWGSC (International Wheat Genome Sequencing Consortium (IWGSC), 2014) RefSeq v1.0 annotation (**Supplementary Table 5**). Candidate genes were prioritized based on physical proximity to significant SNPs and functional annotation associated with carbohydrate metabolism, cell wall biosynthesis, transport, and polysaccharide remodeling. To further prioritize biologically relevant candidates, gene expression profiles were examined using publicly available RNA-seq datasets available through the Wheat Expression Browser. Genes showing substantial expression in developing grain tissues, particularly the endosperm, aleurone layer, and seed coat, were considered stronger functional candidates (**Supplementary Table 6**).

For **WE pentosan** content, candidate genes were identified on chromosomes 1B, 7A, and 7B. On chromosome 1B, the significant marker region contained a bidirectional sugar transporter SWEET gene (TraesCS1B01G066900), suggesting a possible role in carbohydrate allocation and soluble polysaccharide accumulation. This gene was predominantly expressed in the starchy endosperm and associated with SNP marker BS00087787_51 (**Supplementary Table 6**).

The strongest enrichment of candidate genes was detected on chromosome 7A, where a cluster of cellulose synthase-like genes was identified, including TraesCS7A01G394100, within which one significant SNP marker was directly located. Another significant SNP marker was positioned within the glycosyltransferase-related gene TraesCS7A01G394500 on chromosome 7A. This gene was expressed across developing grain tissues, including the whole endosperm, starchy endosperm, aleurone layer, and seed coat, and was associated with SNP marker AX-108918650 (**Supplementary Table 6**). Nearby genes included galactan beta-1,4-galactosyltransferase GALS1 and additional glycosyltransferase-related proteins, indicating that this genomic region may contribute to arabinoxylan and hemicellulose biosynthesis.

Furthermore, TraesCS7A01G395300 (GENE-4898_208) on chromosome 7A was predominantly expressed in the aleurone layer and seed coat, whereas TraesCS7B01G296500 (AX-95148416) on chromosome 7B was expressed throughout developing grain tissues. In addition, TraesCS7A01G393600 and TraesCS7B01G295600 showed particularly high expression in the aleurone layer, further supporting the importance of this genomic region for cell wall polysaccharide biosynthesis (**Supplementary Table 6**).

In the vicinity of β-glucan-associated loci, candidate genes were detected on chromosomes 2B, 4B, and 6B. Several annotated genes encoded proteins involved in cell wall remodeling and carbohydrate metabolism, including xyloglucan endotransglucosylase/hydrolase and endoglucanase genes on chromosome 6B (TraesCS6B01G368300, TraesCS6B01G368000 both associated with wsnp_Ex_c1276_2445537). These enzymes may be associated with β-glucan turnover and polysaccharide restructuring. In addition, the TG0010b marker was located within the *Rht1* gene on chromosome 4B, whereas the chromosome 2B region contained several clustered genes encoding transport-related proteins. The TraesCS4B01G043000 gene, associated with TG0010b on chromosome 4B, showed the highest expression in the seed coat and aleurone layer (**Supplementary Table 6**).

Near **fructan**-associated SNPs, annotated genes were identified on chromosomes 2B, 2D, 3A, 3D, and 7A. The chromosome 2B region included a beta-glucosidase gene (TraesCS2B01G591900), while chromosome 3A contained genes encoding beta-fructofuranosidase 1, acid invertase 1, and mannan endo-1,4-beta-mannosidase-like proteins, all of which may contribute to fructan metabolism and carbohydrate turnover. Genes associated with sugar transport and fructan degradation were predominantly identified on chromosomes 3A and 7A. The significant SNP AX-158567449 on chromosome 7A was located near a cluster of GH32 family genes associated with fructan degradation. The region contained several beta-fructofuranosidase 1 genes (TraesCS7A01G009600, TraesCS7A01G009700, and TraesCS7A01G009200) as well as acid invertase 1 genes (TraesCS7A01G009500 and TraesCS7A01G009800). Regarding genes associated with fructan content, TraesCS7A01G009800 (AX-158567449, Chr. 7A) was highly expressed in the seed coat. TraesCS7A01G009500 was predominantly expressed in the whole endosperm and aleurone layer, whereas TraesCS7A01G009600 showed the highest expression in the starchy endosperm. Additional highly expressed genes within the GH32 cluster included TraesCS7A01G009400, TraesCS7A01G009200, and TraesCS7A01G009100, particularly in the aleurone layer and/or seed coat. TraesCS2D01G248900 (TA003872-0714, Chr. 2D) was highly expressed throughout developing grain tissues, with the highest expression detected in the aleurone layer. TraesCS2D01G273300 (AX-89640158), located near the strongest fructan-associated locus (PVE = 13.3%), also showed substantial expression in grain tissues, supporting its candidacy (**Supplementary Table 6**).

Additional ATPase- and cytochrome-associated transport proteins were also identified within this interval. Together, these results suggest that the chromosome 7A region may represent a major hotspot associated with fructan turnover and carbohydrate transport. Near the strongest fructan-associated marker on chromosome 2D (AX-89640158), two functionally relevant annotated genes were identified: a glutathione S-transferase theta 3-like gene (TraesCS2D01G273800), putatively associated with metabolic processes, and an importin-like protein gene (TraesCS2D01G273300), potentially involved in intracellular transport. However, TraesCS2D01G273800 showed no detectable expression in the examined developing grain tissues, whereas TraesCS2D01G273300 displayed substantial expression. Therefore, the expression data provide stronger support for TraesCS2D01G273300 as the more plausible functional candidate underlying this association. Overall, these analyses revealed that multiple fiber-associated genomic regions contain genes with putative functions related to carbohydrate biosynthesis, sugar transport, cell wall organization, and metabolic regulation, supporting the biological relevance of the identified GWAS loci.

## 4 Discussion

### 4.1 Genetic differentiation and diagnostic marker identification in spelt wheat

Genetic differentiation between spelt wheat (*Triticum spelta* L.) and common wheat (*Triticum aestivum* L.) can be effectively achieved using various molecular markers.

In our study, we assembled a diverse panel of 175 spelt, 80 bread wheat, and 13 wild hexaploid genotypes with a particular focus on spelt germplasm. Our analyses based on high-density 25K SNP array data confirmed that spelt represents a genetically distinct gene pool compared to modern bread wheat cultivars, consistent with previous reports (Siedler et al., 1994; Bertin et al., 2001; Blatter et al., 2002; Liu et al., 2018; Müller et al., 2018). Kinship analysis clearly demonstrated the genetic differentiation between bread wheat and spelt genebank accessions (**Figure 2**). Furthermore, the analysis enabled the identification of lines likely originating from historical hybridization events between spelt and modern bread wheat varieties. These accessions showed closer genetic relationships to modern wheat cultivars rather than to the relatively more homogeneous spelt cluster (**Figure 2**).

Gene-specific analyses also play an important role in distinguishing the two species. The Q locus, located on chromosome 5A, is a key domestication gene influencing spike morphology and threshability. Differences in allelic forms (Q vs. q) and their interactions with other genes significantly contribute to the development of the typical spelt phenotype (Luo et al., 2000; Sormacheva et al., 2015). We identified 16 spelt-specific diagnostic SNP markers, seven of which were clustered within the domestication-related Q locus (**Figure 3**, **Supplementary Table 3**). Previous studies demonstrated that PCR-based approaches, including allele-specific PCR and Kompetitive Allele Specific PCR (KASP), enable the development of highly precise diagnostic marker systems for wheat genotyping. In light of these findings, the conversion of the identified diagnostic SNP markers into KASP assays may facilitate the rapid and cost-effective identification of spelt genetic background in breeding materials and genebank accessions.

### 4.2 Fiber-related SNP markers in spelt

It was shown that there is no significant difference in the total dietary fiber content of spelt and bread wheat grains, despite the difference in thousand grain weight, with spelt having larger kernels. Our previous study (Rakszegi et al., 2025) demonstrated that, in terms of specific dietary fiber components, spelt generally contains lower levels of water-extractable (WE) pentosans, fructans, and β-glucans compared to bread wheat.

Species had a strong effect on soluble fiber composition, particularly on β-glucan levels, whereas grain size influenced only β-glucan and fructan content in bread wheat. In addition, variation in these fiber-related traits was generally narrower in spelt, indicating a more limited range of diversity compared to bread wheat. These observations suggest that although genetic variability is present in spelt, the fluctuation of WE-pentosan, fructan, and β-glucan content is lower than in modern bread wheat cultivars. The relatively lower fructan content observed in spelt may be advantageous for specialized dietary applications, including low-FODMAP products, whereas the reduced β-glucan levels may contribute less to the health-promoting effects commonly associated with soluble dietary fibers. Overall, these findings indicate that spelt differs from bread wheat not only in the absolute levels of major fiber components but also in the extent of phenotypic variability associated with these traits.

The reviewed studies consistently demonstrate that the dietary fiber content of wheat, particularly arabinoxylan (AX), β-glucan, and related fractions, is under strong genetic control and can be effectively targeted through molecular breeding approaches (Gebruers et al., 2010). A key conclusion across the literature is that substantial genetic variation exists for these traits, and numerous quantitative trait loci (QTLs), single nucleotide polymorphisms (SNPs), and Kompetitive Allele-Specific PCR (KASP) markers have been identified and validated, enabling more precise selection for improved fiber composition (Lovegrove et al., 2020; Ibba et al., 2021).

One of the most important and repeatedly confirmed findings is the central role of chromosome 1B, particularly the long arm (1BL), in the regulation of arabinoxylan (AX) content. Studies summarized by Prins and Kosik (2023), together with the experimental work of Lovegrove et al. (2020), identified a major QTL originating from the Yumai-34 cultivar that is strongly associated with elevated AX levels. The marker AX-94524314 (also referred to as BA00789946) showed a robust association with relative viscosity, an indirect indicator of AX content, and was successfully converted into a KASP assay. Additional chromosome 1B markers, including AX-94618000 and loci reported by Ibba et al. (2021), further confirmed the importance of this genomic region, with some loci explaining up to ~20% of the phenotypic variation in water-extractable AX (WE-AX).

In the present study, 24, 10, 17, and 20 significant SNP markers were identified for total pentosan (TOT-pentosan/arabinoxylan), water-extractable pentosan (WE-pentosan), β-glucan, and fructan content, respectively, using a significance threshold combined with PVE values above 3%. The most significant WE-pentosan-associated markers were located on chromosomes 1B, 2A, 7A, and 7B, corresponding well with previously described chromosomal regions involved in arabinoxylan biosynthesis. In particular, our candidate gene analysis highlighted several biologically relevant loci on chromosome 7A, including cellulose synthase-like genes, glycosyltransferase-related genes, and galactan beta-1,4-galactosyltransferase GALS1-associated regions, supporting the involvement of this interval in hemicellulose and arabinoxylan biosynthesis (**Supplementary Table 5**). On chromosome 1B, the significant marker interval also contained a bidirectional sugar transporter SWEET gene, suggesting a potential role in carbohydrate allocation and soluble polysaccharide accumulation (**Supplementary Table 5**). Expression profiling provided additional support for these candidates: TraesCS1B01G066900 was predominantly expressed in the starchy endosperm, TraesCS7A01G394500 was expressed across developing grain tissues, and TraesCS7A01G395300 showed its strongest expression in the aleurone layer and seed coat (**Supplementary Table 6**).

Beyond chromosome 1B, several studies identified important loci across the wheat genome associated with fiber composition. Marcotuli et al. (2015) detected 37 significant marker-trait associations (MTA) linked to AX content in tetraploid wheat, distributed across 19 QTL regions. Many of these loci were located near genes involved in AX biosynthesis and cell wall remodeling, including glycosyl hydrolases, glucan synthases, cellulose synthases, and β-1,4-endoglucanases, highlighting the biological relevance of these genomic regions. Similarly, Jiang et al. (2024) identified stable, environment-independent MTAs for total, water-extractable, and water-unextractable AX on chromosomes 1B, 2A, 5D, and 7A. The chromosome 7A locus detected in the present study was approximately 131 Mb from their reported locus, indicating that these represent distinct genomic regions. Our chromosome 1B locus was approximately 145 Mb from previously reported AX-associated regions (Bordes et al., 2011; Quraishi et al., 2011; Ibba et al., 2021; Jiang et al., 2024), whereas the chromosome 2A locus was only approximately 15 Mb from the stable multi-trait locus reported by Jiang et al. (2024), suggesting that they may represent the same or closely linked genomic region. Jiang et al. (2024) did not identify a WE-pentosan-associated locus on chromosome 7B. However, Marcotuli et al. (2015) reported a genomic region associated with high arabinoxylan content in durum wheat close to the chromosome 7B centromere. Interestingly, the sTable 7B marker identified in the present study is also located within this centromeric region, providing additional support for the importance of this genomic interval in determining arabinoxylan content.

Genome-wide association studies (GWAS) have substantially expanded the understanding of the genetic architecture underlying wheat fiber composition. Lee et al. (2023) identified eight significant SNP markers in hexaploid wheat, with major loci located on chromosomes 1BL, 4BL, and 5DL. These markers were associated with genes involved in regulatory and stress-response pathways, including F-box proteins and transcription factors. Importantly, their study demonstrated that the accumulation of favorable alleles across multiple loci had an additive effect, significantly increasing arabinoxylan (AX) content.

In addition to AX, several studies also investigated related fiber components such as β-glucan and feruloylated arabinoxylan (FAX). Zhan et al. (2019) identified 111 marker–trait associations linked to FAX content, with the strongest and most stable loci located on chromosomes 6A and 6B. Their results further demonstrated that FAX accumulation was strongly associated with the number of favorable alleles, highlighting the importance of marker stacking and haplotype-based selection strategies in breeding programs. More recent validation studies, such as Hernandez-Espinosa et al. (2024), confirmed the practical applicability of previously identified loci. By validating KASP markers targeting major QTLs on chromosomes 1BL and 6BS, the authors demonstrated that combined marker usage substantially increased the explained phenotypic variance, supporting their effectiveness in marker-assisted breeding.

Mitchell et al. (2026) identified a chromosome 6B SNP associated with a mislocalized peroxidase variant (PER1-v) that cannot cross-link ferulic acids, thereby reducing arabinoxylan dimerization and increasing WE-AX content. This marker may therefore facilitate the identification of high-WE-AX wheat germplasm.

Our results are consistent with these observations and further support the polygenic nature of wheat fiber composition. **Figure 5** revealed that favorable haplotype alleles frequently accumulated together in genotypes exhibiting superior fiber-related phenotypes, indicating a clear additive effect among loci associated with pentosan content.

The favorable, wheat-specific allele of marker AX-108804304 appears to be essential for achieving high WE-pentosan content (**Table 2**, **Figure 5**). However, our results also suggest that the presence of additional favorable wheat alleles may further contribute to increased pentosan levels, indicating that the trait is likely controlled by the combined effects of multiple loci. These patterns suggest that the simultaneous selection of multiple favorable alleles may substantially improve dietary fiber composition in wheat breeding programs.

GWAS studies indicate that β-glucan content in wheat is a genetically complex trait controlled by multiple loci distributed across the genome, although fewer stable markers have been identified compared with arabinoxylan-related traits. According to Prins and Kosik (2023), β-glucan-associated QTLs have been reported in both tetraploid and hexaploid wheat, but their effects are generally moderate and often environment-dependent. Marcotuli et al. (2016) further refined this genetic architecture in tetraploid wheat by identifying significant SNP markers across chromosomes 1A, 2A, 2B, 5B, and 7A. Several of these loci were linked to genes involved in carbohydrate metabolism, including endo-β-1,4-glucanase, β-amylase, β-glucosidase, fructan 1-exohydrolase, and 1,4-beta-xylanase, suggesting that β-glucan accumulation is influenced not only by specific biosynthetic pathways but also by broader carbohydrate metabolic processes. Additionally, studies on wild relatives, such as *Aegilops biuncialis* and wheat-barley introgression lines identified novel QTLs on chromosomes 1M, 4M, 5M, 7M and 7H suggesting that these species may serve as valuable genetic resources for enhancing β-glucan content of wheat (Cseh et al., 2013; Rakszegi et al., 2017; Türkösi et al., 2018).

Our results are consistent with these observations. Significant SNPs associated with β-glucan content were identified on chromosomes 2A, 2B, 4B, 5A, 5B, 6B, and 7A, with the strongest associations detected on chromosomes 2B, 4B, and 6B (**Table 2**, **Supplementary Table 5**). Candidate gene analysis further supported the biological relevance of these loci. In particular, the chromosome 6B region contained genes encoding xyloglucan endotransglucosylase/hydrolase and endoglucanase (TraesCS6B01G368300 and TraesCS6B01G368000), both involved in cell wall remodeling and carbohydrate metabolism. In addition, the significant TG0010b marker on chromosome 4B was located within the *Rht1* gene, suggesting a possible relationship between plant developmental regulation and β-glucan-associated grain composition. The favorable, wheat-specific alleles of both BS00067878_51 and TG0010b appeared to be essential for achieving the highest β-glucan contents (**Table 2**, **Figure 5**). The TraesCS4B01G043000 gene associated with TG0010b was expressed across the examined developing grain tissues, although its expression was markedly higher in the seed coat and aleurone layer than in the whole and starchy endosperm (**Supplementary Table 6**). However, the presence of additional favorable wheat alleles at other loci may further enhance β-glucan accumulation, suggesting that the trait is controlled by the combined effects of multiple genetic factors.

Overall, these findings support that β-glucan content is a polygenic trait influenced by multiple genomic regions, most of which have moderate effects. Despite the identification of several genomic regions associated with β-glucan content, the relatively limited variation observed within cultivated wheat suggests that the potential for substantially increasing β-glucan content through selection within the existing wheat gene pool may be constrained. Therefore, the incorporation of novel alleles from wild relatives and related germplasm may represent an important strategy for broadening the genetic base and enhancing β-glucan content in future wheat breeding programs.

Genome-wide association studies investigating fructan content in wheat and related species indicate that **fructan** accumulation is a complex quantitative trait controlled by multiple genomic regions and strongly influenced by environmental conditions, particularly drought stress. Across studies, numerous MTAs, SNPs, and QTLs have been identified, although their individual effects are generally moderate and often environment-dependent. One of the most consistently reported genomic regions is located on chromosome 7A. Gaur et al. (2022) identified 93 MTAs across 39 genomic regions, highlighting major loci on chromosomes 4A and 7A containing key fructan biosynthesis genes, including *1-SST*, *1-FFT*, and *6-SFT*. Huynh et al. (2008, 2012) also reported major QTLs on chromosomes 7A and 6D associated with grain fructan content and demonstrated that sequence variation within the fructan biosynthetic gene cluster, including the *Ta1-SST* region, contributes substantially to fructan accumulation. Additional GWAS studies further confirmed the polygenic architecture of the trait across multiple chromosomes (Zeng et al., 2020; Bajgain et al., 2023), while work in barley also demonstrated that polymorphism in core fructan pathway genes on chromosome 7H, directly affects fructan composition (Matros et al., 2021). Our results are consistent with these observations. Fructan-associated SNPs were detected on chromosomes 2A, 2B, 2D, 3A, 3B, 3D, 5A, and 7A, with the strongest signals identified on 2B, 2D, 3A, 3D, and 7A (**Table 2**, **Supplementary Table 5**). Candidate gene analysis revealed several biologically relevant loci associated with carbohydrate metabolism and sugar transport. On chromosome 2B, the significant marker region was located near a *beta-glucosidase* gene (*TraesCS2B01G591900*), while the 2D regions contained candidate genes related to intracellular transport and metabolic regulation, including *syntaxin* (*TraesCS2D01G248900*) and a glutathione S-transferase theta 3-like gene (*TraesCS2D01G273800*). A particularly notable signal was identified on chromosome 3A, where multiple significant SNPs co-localized with genes encoding sugar transporters and a *mannan endo-1,4-beta-mannosidase-like* protein (*TraesCS3A01G274200*), suggesting a role in carbohydrate turnover and remobilization during grain development. The strongest biological support was observed on chromosome 7A. The significant marker AX-158567449 was located adjacent to a dense cluster of GH32 family genes associated with fructan degradation, including multiple *beta-fructofuranosidase 1* and *acid invertase 1* genes (**Table 2**, **Supplementary Table 5**). This region also contained additional transport-related genes, including ATPase- and cytochrome-associated transport proteins. Together, these findings indicate that the chromosome 7A interval represents a major genomic hotspot associated with fructan turnover, degradation, and carbohydrate transport in wheat grain.

Expression data provided additional support for several candidate genes: TraesCS2D01G248900 was highly expressed across developing grain tissues, particularly in the aleurone layer, while TraesCS7A01G009500, TraesCS7A01G009600, and TraesCS7A01G009800 displayed complementary tissue-specific expression patterns, with predominant expression in the whole endosperm and aleurone layer, starchy endosperm, and seed coat, respectively (**Supplementary Table 6**). The wheat-specific alleles of both AX-89640158 and AX-108781921 appeared to be required for achieving the highest fructan contents (**Table 2**, **Figure 5**). At the strongest association on chromosome 2D, the importin-like gene TraesCS2D01G273300, located near the SNP marker AX-89640158, showed substantial expression across developing grain tissues, whereas the neighboring gene TraesCS2D01G273800 was not detectably expressed. This contrasting expression pattern provides stronger support for TraesCS2D01G273300 as the more plausible functional candidate underlying this locus.

Nevertheless, the contribution of additional wheat-specific alleles indicates that fructan accumulation is likely regulated by the cumulative effects of multiple loci. From a nutritional perspective, however, the generally lower fructan content observed in spelt may be considered advantageous.

In summary, current evidence indicates that fructan content is controlled by multiple loci distributed across the wheat genome, with particularly important recurrent regions on chromosomes 7A, 6D, and 4A. Our results further highlight chromosome 3A as an additional relevant genomic region. Although the effects of individual loci are generally moderate, the combined action of multiple favorable alleles likely contributes substantially to fructan accumulation. These findings provide a strong basis for marker-assisted selection and future breeding aimed at modifying wheat grain fructan composition.

Although breeding specifically aimed at reducing fructan content in wheat or spelt has not been reported, whole-grain wheat generally contains 0.7–2.9% fructan (Huynh et al., 2008, 2012), whereas minimum levels of 0.4–0.7% have been reported for raw wheat and spelt (Marín-Sanz et al., 2022). The lower value of 0.29% observed here therefore highlights spelt as a promising resource for low-FODMAP breeding. Because fructans contribute to stress tolerance, genetic reduction may be constrained; however, sourdough fermentation can further lower fructan levels and improve tolerance in patients with IBS (Varney et al., 2017).

Taken together, these findings indicate that dietary fiber composition in wheat is controlled by a complex genetic architecture involving multiple loci related to carbohydrate metabolism, cell wall biosynthesis, and sugar transport. While several of the identified genomic regions co-localize with previously reported QTLs, others may represent additional loci contributing to fiber-related trait variation, particularly in spelt wheat. The results also highlight the value of spelt as a distinct genetic resource for studying grain fiber composition and provide a useful basis for future functional validation of candidate genes and the genetic improvement of wheat fiber-related quality traits.

## 5 Conclusion

The study provides new insights into the genetic architecture of dietary fiber composition in spelt and bread wheat through comprehensive genome-wide association analyses of water-extractable pentosans (arabinoxylan), β-glucan, and fructan. While the genetic basis of these traits has been extensively investigated in bread wheat, comparable high-resolution analyses in spelt have remained limited. The identification of stable marker–trait associations across multiple years, together with candidate genes involved in cell wall biosynthesis, carbohydrate metabolism, and sugar transport, advances the understanding of fiber-related trait regulation in wheat. In addition, the discovery of 16 spelt-specific diagnostic SNP markers provides useful tools for the reliable discrimination of genebank accessions and highlights the unique genetic potential of spelt within the wheat gene pool.

From a breeding perspective, the identified SNP markers represent valuable resources for marker-assisted selection targeting fiber-related quality traits. The stability of several loci across environments further supports their practical applicability in breeding programs. Moreover, the detection of functionally relevant candidate genes may facilitate the future development of gene-based breeding strategies following functional validation.

The identified genomic regions associated with fructan, β-glucan, and arabinoxylan metabolism may also contribute to the development of wheat varieties with improved nutritional and functional properties, including low-FODMAP products and enhanced prebiotic potential. Furthermore, the utilization of spelt genetic diversity may support sustainable wheat improvement and low-input agricultural systems. Overall, the study connects genomic discovery with practical breeding applications and provides new molecular resources for the improvement of nutritional grain quality in wheat.

## Data Availability

The datasets and name of accession numbers presented in this study can be found in **Supplementary Materials**. **Supplementary Table 7** includes raw biochemical datas.

## Supplementary material

Supplementary Figure 1 Variance of the first three principal components in principal component analysis.

Supplementary Figure 2 Boxplots showing significant differences in fiber content associated with different alleles identified in the GWAS panel. “Spelt” indicates that the corresponding allele is more common in spelt than in bread wheat.

Supplementary Table 1 List of studied genotypes.

Supplementary Table 2 Mean, standard deviation, minimum and maximum values of thousand kernel weight and fiber components (total (TOT) and water extractable (WE) pentosan, β-glucan, fructan) in selected spelt (n=50) and bread wheat (n=50) genotypes harvested in 2021 and 2022.

Supplementary Table 3 Diagnostic SNP markers differentiating spelt and bread wheat and the distribution of spelt-associated alleles among bread wheat cultivars.

Supplementary Table 4 Full set of markers on dietary fibers with -log10(p)>3.

Supplementary Table 5 Annotated shortlist of candidate genes identified within ±1 Mb intervals of significant SNP markers associated with fiber-related traits in wheat (WE-pentosan, β-glucan and fructan content).

Supplementary Table 6 Expression profiles of candidate genes associated with dietary fibers (WE-pentosan, β-glucan and fructan content).
