# Supplementary material for: Genome-wide association analysis of fiber-related traits and identification of diagnostic SNP markers differentiating spelt wheat (Triticum spelta L.) from bread wheat
Source: Front Plant Sci. 2026 Aug 7;17:1907921. doi: 10.3389/fpls.2026.1907921 (PMC13493604; doi:10.3389/fpls.2026.1907921)
Supplement: Supplementary file 1 [file DataSheet1.pdf]

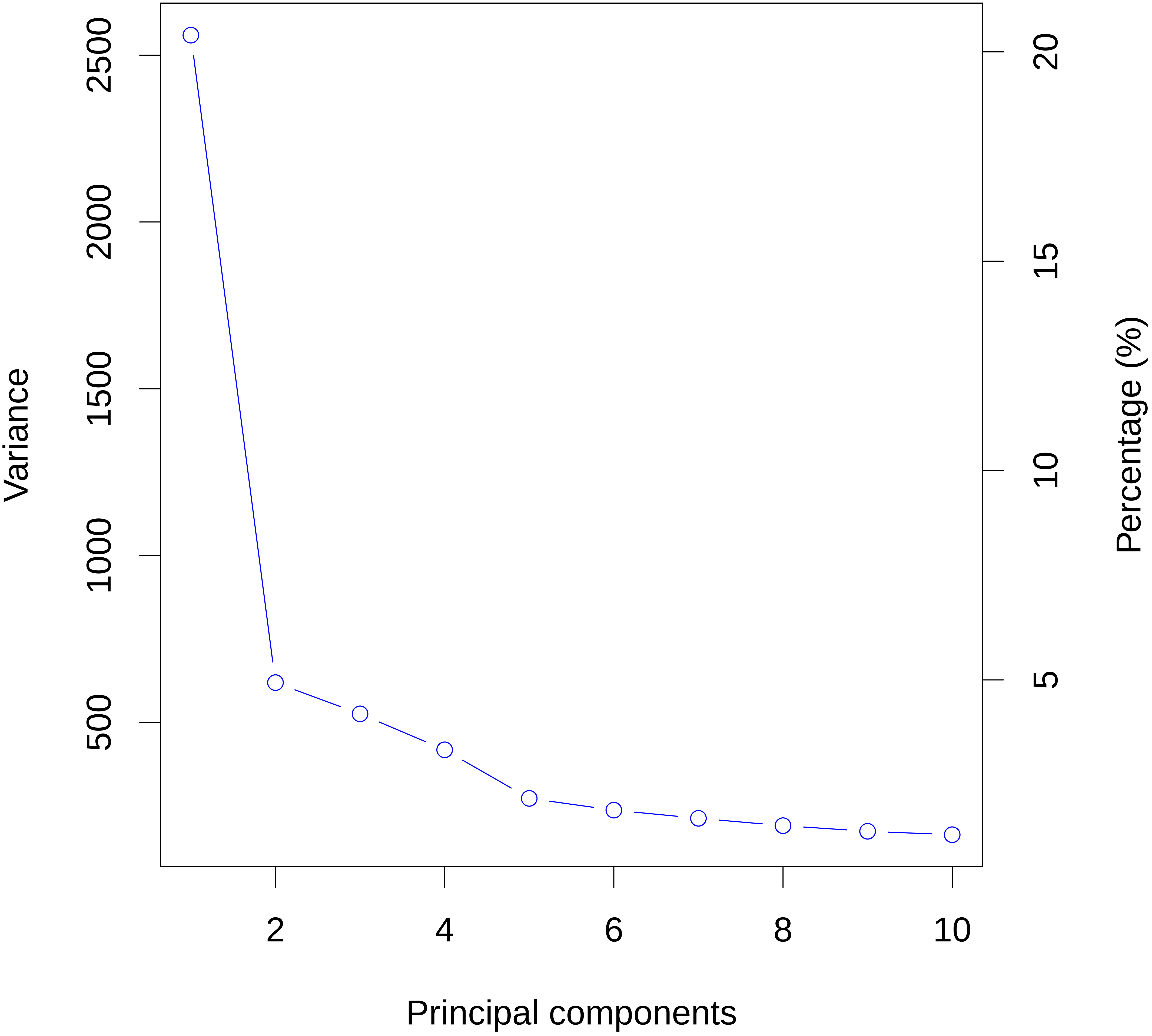
Variance
2500
2000
1500
1000
500
Percentage (%)
20
15
10
5
2
4
6
8
10
Principal components
