# Supplementary material for: Genome-wide association analysis of fiber-related traits and identification of diagnostic SNP markers differentiating spelt wheat (Triticum spelta L.) from bread wheat
Source: Front Plant Sci. 2026 Aug 7;17:1907921. doi: 10.3389/fpls.2026.1907921 (PMC13493604; doi:10.3389/fpls.2026.1907921)
Supplement: Supplementary file 2 [file Table1.docx]

Supplementary Table 1. List of studied genotypes

| **Code** | **Pedigree** | **Growing**  **habit** | **Origin** | **Genebank** | **Identifier**  **of the genotypes**  **in Martonvásár**  **genebank** | **Type of**  **spike** | **Colour**  **of**  **Spike** | **Form**  **of**  **spike** | **Selected 100 samples based on SNP analysis** | |
| --- | --- | --- | --- | --- | --- | --- | --- | --- | --- | --- |
| BW01 | YUMAI-34 | F | CN | Research Institute of Agricultural Sciences of Dong Ying, China | PAz56063SID0 | A | W | BW | **X** |  |
| BW02 | UKRAINKA | W | UA | National Centre for Plant Genetic Resources of Ukraine, Ukraine | NGBAB035227  PAz18261SID0 | A | W | BW |  |  |
| BW03 | SOLSTICE | W | GB | Rothamsted Research, Harpenden, UK | PAz61978SID0 | AL | W | BW | **X** |  |
| BW04 | KORELI | W | FR | INRA UMR Amelioration et Sante des Plantes, France | PAz62252SID0 | A | W | BW | **X** |  |
| BW05 | LONA | F | CH | Delley Samen und Pflanzen AG, Delley, Switzerland | NGBAB034499  PAz10747SID0 | A | W | BW | **X** |  |
| BW06 | MV-MARSALL | W | HU | HUN-REN ATK MGI, Martonvasar, Hungary | NGBAB035121  PAz38586SID1 | A | W | BW | **X** |  |
| BW07 | NX02Y4530 | W | USA | USDA-ARS University of Nebraska, Lincoln, USA | PAz58330SID1 | A | W | BW |  |  |
| BW08 | MULAN | W | DE | BOKU, Institute for Agrobiotechnology, Tulln, Austria | PAz59957SID0 | AL | W | BW | **X** |  |
| BW09 | MV-LUCILLA | W | HU | HUN-REN ATK MGI, Martonvasar, Hungary | NGBAB035114  PAz23464SID9 | A | W | BW | **X** |  |
| BW10 | MOULIN | W | GB | INRA UMR Amelioration et Sante des Plantes, France | PAz11691SID0 | AL | W | BW | **X** |  |
| BW11 | VALORIS | W | FR | INRA UMR Amelioration et Sante des Plantes, France | PAz56728SID0 | AL | W | BW | **X** |  |
| BW12 | ZVEZDA | W | CS | Agroseme Pannonija Subotica, Subotica, Serbia | NGBAB035240  PAz19106SID0 | AL | W | BW |  |  |
| BW13 | THESEE | W | FR | INRA UMR Amelioration et Sante des Plantes, France | PAz1631SID1 | AL | W | BW |  |  |
| BW14 | BISCAY | W | DE | PZO Saat GmbH - Pflanzenzucht Oberlimpurg, Germany | PAz56540SID0 | AL | W | BW | **X** |  |
| BW15 | MAGDALENA | W | FR | INRA UMR Amelioration et Sante des Plantes, France | PAz55369SID0 | AL | W | BW |  |  |
| BW16 | BLE-DES-DOMES | W | FR | INRA UMR Amelioration et Sante des Plantes, France | PAz56717SID0 | AL | W | BW |  |  |
| BW17 | AMADEUS | W | AT | Probstdorfer Saatzucht GesmbH & Co KG, Probstdorf, Austria | PAz1923SID0 | AL | W | BW | **X** |  |
| BW18 | MOMCHIL | W | BG | IPGR, Institute for Plant Genetic Resources “Konstantin Malkov”, Sadovo, Bulgaria | NGBAB034595  PAz11663SID0 | AL | W | BW | **X** |  |
| BW19 | CUBUS | W | DE | PZO Saat GmbH - Pflanzenzucht Oberlimpurg, Germany | PAz56541SID0 | AL | W | BW | **X** |  |
| BW20 | GK-TISZATAJ | W | HU | Cereal Research Non-Profit Ltd., Szeged, Hungary | PAz17729SID0 | A | W | BW | **X** |  |
| BW21 | SEU-SEUN-27 | W | KR | INRA UMR Amelioration et Sante des Plantes, France | PAz56726SID0 | A | W | BW |  |  |
| BW22 | ATAY-85 | F | TR | CIMMYT, Turkey | PAz2066SID0 | A | W | BW | **X** |  |
| BW23 | MONOPOL | W | DE | IPK-Gatersleben, Genebank Department, Gatersleben, Germany | PAz11670SID0 | AL | W | BW | **X** |  |
| BW25 | KANZLER | W | DE | IPK-Gatersleben, Genebank Department, Gatersleben, Germany | PAz9945SID0 | AL | W | BW | **X** |  |
| BW26 | HEREWARD | W | GB | Nickerson UK Ltd., Limagrain, Woolpit, Bury St Edmunds, Suffolk, UK | PAz7758SID0 | AL | W | BW | **X** |  |
| BW27 | ALBA | W | PL | Danko Hodowla Roślin Sp. z o.o z/s w Choryni, Koscian, Poland | PAz1858SID0 | AL | W | BW | **X** |  |
| BW28 | BEGRA | W | PL | Danko Hodowla Roślin Sp. z o.o z/s w Choryni, Koscian, Poland | PAz2357SID0 | AL | W | BW | **X** |  |
| BW29 | MILLENNIUM | W | US | University Nebraska, Department of Agronomy, Lincoln, USA | PAz13536SID1 | A | W | BW | **X** |  |
| BW30 | STEPHENS | W | US | Oregon State University, Department of Crop and Soil Science, Corvallis, USA | NGBAB035211  PAz16814SID0 | A | W | BW | **X** |  |
| BW32 | KEY | W | US | University Nebraska, Department of Agronomy, Lincoln, USA | PAz10042SID0 | AL | W | BW | **X** |  |
| BW33 | CAMP-REMY | W | FR | INRA UMR Amelioration et Sante des Plantes, France | PAz3110SID0 | AL | W | BW | **X** |  |
| BW34 | NOMADE | W | IT | Experimental Institute of Cereal Research, S. Angelo Lodigiano, Italy (closed)  Council for Agricultural Research and Economics  Research Centre for Cereal and Industrial Crops, CREA,  Vercelli, Italy | PAz46756SID1 | A | W | BW |  |  |
| BW35 | GERONIMO | W | IT | Experimental Institute of Cereal Research, S. Angelo Lodigiano, Italy (closed)  Council for Agricultural Research and Economics  Research Centre for Cereal and Industrial Crops, CREA,  Vercelli, Italy | PAz57968SID0 | A | W | BW |  |  |
| BW36 | MARTONVASARI-17 | W | HU | HUN-REN ATK MGI, Martonvasar, Hungary | NGBAB034556  PAz44755SID2 | AL | W | BW | **X** |  |
| BW37 | CARDINAL | W | US | University Nebraska, Department of Agronomy, Lincoln, USA | PAz3129SID0 | A | W | BW |  |  |
| BW38 | KARL-92 | W | US | Kansas State University, Throckmorton Plant Sciences Center, Manhattan, USA | NGBAB034475  PAz9966SID0 | A | W | BW |  |  |
| BW40 | ISENGRAIN | W | FR | INRA UMR Amelioration et Sante des Plantes, France | PAz8638SID0 | A | W | BW | **X** |  |
| BW41 | BUCK-CATRIEL | F | AR | Asociados Don Mario S.A, Argentina | PAz2784SID0 | A | W | BW | **X** |  |
| BW42 | ILJICSOVKA | W | UA | NII Seselkcii i semenovod pshenici, Kievskaya obl., Ukraine | NGBAB034470  PAz21517SID1 | AL | W | BW | **X** |  |
| BW43 | ALABASSKAJA | W | KZ | Vsesoyuznij-geneticheskij Institute, Odessa, Ukraine | PAz1850SID0 | AL | W | BW | **X** |  |
| BW44 | AURORA | W | RU | Vsesoyuznij-geneticheskij Institute, Odessa, Ukraine | NGBAB034397  PAz2097SID1 | AL | W | BW |  |  |
| BW45 | SPARTANKA | W | RU | Vsesoyuznij-geneticheskij Institute, Odessa, Ukraine | NGBAB035210  PAz16451SID0 | AL | W | BW |  |  |
| BW46 | ANTONIUS | W | AT | BOKU, Institute for Agrobiotechnology, Tulln, Austria | PAz60249SID0 | A | W | BW |  |  |
| BW47 | APACHE | W | FR | INRA UMR Amelioration et Sante des Plantes, France | PAz1966SID0 | AL | W | BW |  |  |
| BW48 | ARINA | W | CH | WBF-Agroscope, Switzerland | PAz74676SID0 | AL | W | BW | **X** |  |
| BW49 | BALKAN | W | CS | Institute of Field and Vegetable Crops, Novi Sad, Serbia | PAz2479SID0 | AL | W | BW |  |  |
| BW50 | DEKAN | W | DE | NORDSAAT Saatzucht GmbH., Langenstein, Germany | PAz47303SID0 | A | W | BW | **X** |  |
| BW51 | POBEDA | W | CS | Institute of Field and Vegetable Crops, Novi Sad, Serbia | NGBAB035177  PAz15544SID0 | AL | W | BW |  |  |
| BW54 | DISPONENT | W | DE | INRA UMR Amelioration et Sante des Plantes, France | PAz4286SID0 | AL | W | BW | **X** |  |
| BW55 | BRUTUS | W | AT | Saatzucht Donau GesmbH & CoKG, Probstdorf, Austria | PAz2739SID0 | A | W | BW | **X** |  |
| BW56 | RENAN | W | FR | INRA UMR Amelioration et Sante des Plantes, France | PAz2995SID0 | A | W | BW |  |  |
| BW57 | GLENLEA | S | CA | North Dakota State University, Academics, Faculty of Plant Pathology, Fargo, USA | PAz6858SID0 | AL | W | BW |  |  |
| BW58 | PANNONIKUS | W | AT | Saatzucht Donau GesmbH & CoKG, Probstdorf, Austria | PAz60506SID1 | A | W | BW | **X** |  |
| BW59 | VANEK | S | DE | KWS LOCHOW GMBH Zuchtstation Wetze, Northheim, Germany | PAz55399SID0 | AL | W | BW | **X** |  |
| BW60 | BREA | W | CZ | Research Institute of Crop Production VÚRV, Prague, Czech Republic | PAz2708SID0 | AL | W | BW | **X** |  |
| BW61 | GK-BEKES | W | HU | Cereal Research Non-Profit Ltd., Szeged, Hungary | PAz53088SID0 | A | W | BW |  |  |
| BW62 | RECITAL | W | FR | INRA UMR Amelioration et Sante des Plantes, France | PAz15808SID0 | AL | W | BW | **X** |  |
| BW63 | VLASTA | W | CZ | Research Institute of Crop Production VÚRV, Prague, Czech Republic | PAz18435SID0 | AL | W | BW |  |  |
| BW64 | WENZEL | W | AT | Saatzucht Donau GesmbH & CoKG, Probstdorf, Austria | NGBAB158282  PAz60506SID0 | AL | W | BW | **X** |  |
| BW65 | LUDWIG | W | AT | University of Osijek, Faculty of Agrobiotechnical Sciences, Osijek, Croatia | PAz10897SID0 | AL | W | BW |  |  |
| BW66 | SIMONIDA | W | CS | Institute of Field and Vegetable Crops, Novi Sad, Serbia | NGBAB158275  PAz63208SID0 | AL | W | BW |  |  |
| BW67 | ESTEVAN | W | AT | University of Osijek, Faculty of Agrobiotechnical Sciences, Osijek, Croatia | PAz67434SID0 | A | W | BW |  |  |
| BW68 | FLAMURA-85 | W | RO | Research Institute for Cereal and Industrial Crops, Fundulea, Romania | NGBAB034444  PAz6215SID0 | A | W | BW |  |  |
| BW69 | MARIS-HUNTSMAN | W | GB | CIMMYT, Turkey | NGBAB034545  PAz11502SID0 | AL | W | BW |  |  |
| BW70 | RIBAND | W | GB | INRA UMR Amelioration et Sante des Plantes, France | PAz15871SID0 | AL | W | BW |  |  |
| BW71 | LYNX | W | GB | INRA UMR Amelioration et Sante des Plantes, France | PAz11082SID0 | AL | W | BW |  |  |
| BW73 | TREMIE | W | FR | INRA UMR Amelioration et Sante des Plantes, France | PAz17958SID0 | AL | W | BW |  |  |
| BW74 | RIALTO | W | GB | John Innes Centre, Norwich, UK | PAz15869SID0 | AL | W | BW | **X** |  |
| BW75 | CAMPARI | W | DE | PZO Saat GmbH - Pflanzenzucht Oberlimpurg, Germany | PAz56597SID0 | AL | W | BW |  |  |
| BW76 | ATLAS-66 | F | US | INRA UMR Amelioration et Sante des Plantes, France | PAz2072SID0 | AL | W | BW | **X** |  |
| BW77 | MV-SUBA | W | HU | HUN-REN ATK MGI, Martonvasar, Hungary | NGBAB035140  PAz24399SID1 | A | W | BW |  |  |
| BW78 | MV-SUVEGES | W | HU | HUN-REN ATK MGI, Martonvasar, Hungary | NGBAB035142  PAz32899SID7 | A | W | BW | **X** |  |
| BW79 | BEZOSTAJA-1 | W | RU | University Nebraska, Department of Agronomy, Lincoln, USA | PAz2427SID1 | AL | W | BW | **X** |  |
| BW80 | BALATON | W | AT | Saatzucht Donau GesmbH & CoKG, Probstdorf, Austria | NGBAB158263  PAz54420SID0 | AL | W | BW | **X** |  |
| BW81 | Big Club 43 (*T.a. compactum*) | W | HU | Australian Grains Genebank, Victoria, Australia | NGBAB033198  PAz101766SID0 | AL | W | WH | **X** |  |
| BW83 | MVGB323 (*T.a. compactum*) | W | HU | University of Hohenheim, Stuttgart, Germany | NGBAB033178  PAz101747SID0 | AL | W | WH | **X** |  |
| BW84 | MVGB324 (*T.a. compactum*) | W | HU | University of Hohenheim, Stuttgart, Germany | NGBAB033179  PAz101748SID0 | AL | W | WH | **X** |  |
| BW85 | MVGB356 (*T.a. compactum*) | W | HU | HUN-REN ATK MGI, Martonvasar, Hungary | NGBAB033211  PAz101776SID0 | A | W | WH |  |  |
| BW86 | MVGB583 (*T.a. compactum*) | W | HU | HUN-REN ATK MGI, Martonvasar, Hungary | NGBAB033432  PAz101984SID0 | AL | W | WH |  |  |
| BW87 | MVGB357 (*T.a. compactum*) | W | HU | HUN-REN ATK MGI, Martonvasar, Hungary | NGBAB033212  PAz101777SID0 | AL | W | WH |  |  |
| BW88 | MVGB322 (*T.a. compactum*) | S | HU | University of Hohenheim, Stuttgart, Germany | NGBAB033177  PAz101746SID0 | AL | W | WH | **X** |  |
| BW89 | MVGB1175 (*T.a. compactum*) | S | HU | Agriculture and Agri-Food Canada, Cereal Research Centre, Manitoba, Canada | NGBAB033911  PAz102449SID0 | AL | W | WH |  |  |
| BW90 | GLUCLUB (*T.a. compactum*) | S | HU | USDA-ARS Plant Introduction Office, Pullman, USA | NGBAB033322  PAz101878SID0 | AL | W | WH | **X** |  |
| BW91 | MVGB1071 (*T.a. macha*) | S | HU | National Biodiversity and Gene Conservation Center, Tapioszele, Hungary | NGBAB033866  PAz102409SID0 | AL | W | WH | **X** |  |
| BW92 | LYALLPUR (*T.a. sphearococcum*) | S | PK | National Biodiversity and Gene Conservation Center, Tapioszele, Hungary | NGBAB033325  PAz101881SID0 | AL | W | WH |  |  |
| BW93 | MVGB657 (*T.a. compactum*) | S | HU | University of California, College of Natural & Agricultural Sciences, Riverside, CA USA | NGBAB033506  PAz102054SID0 | AL | W | WH | **X** |  |
| BW94 | MVGB543 (*T.a. macha*) | W | HU | HUN-REN ATK MGI, Martonvasar, Hungary | NGBAB033394  PAz101947SID0 | A | W | WH |  |  |
| TSP01 | OSTRO | W | CH | University of Hohenheim, Stuttgart, Germany | PAz14860SID0 | AL | R | SP |  |  |
| TSP02 | ROUQUIN | W | BE | University of Hohenheim, Stuttgart, Germany | PAz15952SID0 | AL | R | SP |  |  |
| TSP03 | BOHEMIA | S | CZ | NATUR GOLD FARMS KFT, Szendrő, Hungary | PAz59558SID0 | A | B | SP |  |  |
| TSP04 | GEOFFS-RED-SPELT | F | AU | Australian Grains Genebank, Victoria, Australia | PAz65402SID0 | AL | R | SP |  |  |
| TSP05 | GEOFFS-WHITE-SPELT | F | AU | Australian Grains Genebank, Victoria, Australia | PAz65403SID0 | AL | W | SP |  |  |
| TSP06 | BALMEGG | W | CH | Delley Samen und Pflanzen AG, Delley, Switzerland | PAz2182SID0 | AL | R | SP |  |  |
| TSP07 | HUBEL | W | CH | Delley Samen und Pflanzen AG, Delley, Switzerland | PAz7774SID0 | AL | R | SP |  |  |
| TSP08 | LUEG | W | CH | Delley Samen und Pflanzen AG, Delley, Switzerland | PAz10659SID0 | AL | R | SP |  |  |
| TSP09 | OBERKULMER-ROTKORN | W | CH | Delley Samen und Pflanzen AG, Delley, Switzerland | PAz14337SID0 | AL | R | SP |  |  |
| TSP10 | OSTAR | W | CH | Delley Samen und Pflanzen AG, Delley, Switzerland | PAz14857SID0 | AL | R | SP | **X** |  |
| TSP11 | SERTEL | W | CH | Delley Samen und Pflanzen AG, Delley, Switzerland | PAz16282SID0 | AL | W | SP |  |  |
| TSP12 | CH65384 | W | CH | Delley Samen und Pflanzen AG, Delley, Switzerland | PAz3385SID0 | AL | W | SP |  |  |
| TSP13 | CH65388 | W | CH | Delley Samen und Pflanzen AG, Delley, Switzerland | PAz3386SID0 | AL | W | SP |  |  |
| TSP14 | BAULANDER-SPELZ | W | DE | University of Hohenheim, Stuttgart, Germany | PAz2305SID0 | AL | R | SP |  |  |
| TSP15 | HERCULE | W | BE | University of Hohenheim, Stuttgart, Germany | PAz7514SID0 | AL | W | SP | **X** |  |
| TSP16 | HOLSTENKORN | F | DE | University of Hohenheim, Stuttgart, Germany | PAz7736SID0 | AL | R | SP |  |  |
| TSP17 | SCHWABENKORN | W | DE | University of Hohenheim, Stuttgart, Germany | PAz16225SID0 | AL | R | SP |  |  |
| TSP18 | BA7415 | W | DE | Dr. Alter Pflanzenzucht und Versuchswesen, Felsberg, Germany | PAz54382SID0 | AL | R | SP |  |  |
| TSP19 | BA7418 | W | DE | Dr. Alter Pflanzenzucht und Versuchswesen, Felsberg, Germany | PAz54383SID0 | AL | W | SP |  |  |
| TSP20 | BA7423 | W | DE | Dr. Alter Pflanzenzucht und Versuchswesen, Felsberg, Germany | PAz54384SID0 | AL | R | SP |  |  |
| TSP21 | BA7424 | W | DE | Dr. Alter Pflanzenzucht und Versuchswesen, Felsberg, Germany | PAz54385SID0 | AL | R | SP |  |  |
| TSP22 | FRANCKENKORN | W | DE | PZO Saat GmbH - Pflanzenzucht Oberlimpurg, Schwäbisch Hall , Germany | PAz6288SID0 | AL | R | SP | **X** |  |
| TSP23 | D-7-004-99-01 | W | DE | University of Hohenheim, Stuttgart, Germany | PAz66387SID1 | AL | R | SP |  |  |
| TSP24 | D-7-004-99-02 | W | DE | University of Hohenheim, Stuttgart, Germany | PAz66387SID0 | AL | R | SP |  |  |
| TSP25 | D-7-005-99-01 | W | DE | University of Hohenheim, Stuttgart, Germany | PAz66388SID0 | AL | R | SP |  |  |
| TSP26 | D-7-005-99-04 | W | DE | University of Hohenheim, Stuttgart, Germany | PAz66388SID1 | AL | R | SP |  |  |
| TSP27 | D-7-005-99-06 | W | DE | University of Hohenheim, Stuttgart, Germany | PAz66388SID2 | AL | R | SP | **X** |  |
| TSP28 | D-7-010-99-02 | W | DE | University of Hohenheim, Stuttgart, Germany | PAz66391SID0 | AL | R | SP |  |  |
| TSP29 | D-7-014-98-01 | W | DE | University of Hohenheim, Stuttgart, Germany | PAz66395SID0 | AL | R | SP |  |  |
| TSP30 | D-7-014-99-02 | W | DE | University of Hohenheim, Stuttgart, Germany | PAz66392SID0 | AL | R | SP |  |  |
| TSP31 | D-7-014-99-03 | W | DE | University of Hohenheim, Stuttgart, Germany | PAz66392SID1 | AL | R | SP |  |  |
| TSP32 | D-7-019-99-02 | W | DE | University of Hohenheim, Stuttgart, Germany | PAz66394SID0 | AL | R | SP |  |  |
| TSP33 | COSMOS | W | BE | Research Center Agronomiques De Gembloux, Gembloux, Belgium | PAz56603SID0 | AL | R | SP |  |  |
| TSP34 | POEME | W | BE | Research Center Agronomiques De Gembloux, Gembloux, Belgium | PAz56604SID0 | AL | R | SP | **X** |  |
| TSP35 | REDOUTE | W | BE | Research Center Agronomiques De Gembloux, Gembloux, Belgium | PAz56605SID0 | AL | W | SP |  |  |
| TSP36 | RESSAC | W | BE | Research Center Agronomiques De Gembloux, Gembloux, Belgium | PAz56601SID0 | AL | R | SP | **X** |  |
| TSP37 | SPY | W | BE | Research Center Agronomiques De Gembloux, Gembloux, Belgium | PAz56600SID0 | AL | R | SP | **X** |  |
| TSP38 | STONE | W | BE | Research Center Agronomiques De Gembloux, Gembloux, Belgium | PAz56602SID0 | AL | R | SP |  |  |
| TSP39 | OKO-10 | W | HU | Szentmiklósi Gabona Kft., Törökszentmiklós, Hungary | PAz48477SID0 | AL | R | SP | **X** |  |
| TSP40 | MV-MARTONGOLD | W | HU | HUN-REN ATK MGI, Martonvasar, Hungary | PAz50823SID2 | AL | R | SP | **X** |  |
| TSP41 | OBERKULMER/BAULANDER-SPELZ_1 | W | HU | HUN-REN ATK MGI, Martonvasar, Hungary | PAz50826SID6 | AL | R | SP | **X** |  |
| TSP43 | OBERKULMER/ROUQUIN | W | HU | HUN-REN ATK MGI, Martonvasar, Hungary | PAz50824SID4 | AL | R | SP |  |  |
| TSP44 | OBERKULMER/SCHWABENKORN_1 | W | HU | HUN-REN ATK MGI, Martonvasar, Hungary | PAz50825SID3 | AL | R | SP |  |  |
| TSP45 | OBERKULMER/SCHWABENKORN_2 | W | HU | HUN-REN ATK MGI, Martonvasar, Hungary | PAz50825SID2 | AL | R | SP |  |  |
| TSP46 | OBERKULMER/SCHWABENKORN_3 | W | HU | HUN-REN ATK MGI, Martonvasar, Hungary | PAz50825SID7 | AL | R | SP |  |  |
| TSP47 | OBERKULMER/SCHWABENKORN_4 | W | HU | HUN-REN ATK MGI, Martonvasar, Hungary | PAz50825SID5 | AL | R | SP |  |  |
| TSP49 | OBERKULMER/SERTEL_1 | W | HU | HUN-REN ATK MGI, Martonvasar, Hungary | PAz50822SID11 | AL | R | SP | **X** |  |
| TSP50 | OBERKULMER/SERTEL_2 | W | HU | HUN-REN ATK MGI, Martonvasar, Hungary | PAz50822SID10 | AL | R | SP |  |  |
| TSP51 | TSP04-09 | W | HU | HUN-REN ATK MGI, Martonvasar, Hungary | PAz50824SID1 | AL | R | SP | **X** |  |
| TSP52 | MV-VITALGOLD | W | HU | HUN-REN ATK MGI, Martonvasar, Hungary | PAz50822SID5 | AL | R | SP | **X** |  |
| TSP53 | TSP06-10 | W | HU | HUN-REN ATK MGI, Martonvasar, Hungary | PAz50822SID7 | AL | R | SP |  |  |
| TSP54 | TSP07-09 | W | HU | HUN-REN ATK MGI, Martonvasar, Hungary | PAz50822SID12 | AL | R | SP |  |  |
| TSP55 | MVGB139 | F | HU | HUN-REN ATK MGI, Martonvasar, Hungary | NGBAB033078  PAz77104SID0 | AL | W | SP | **X** |  |
| TSP56 | MVGB140 | F | HU | HUN-REN ATK MGI, Martonvasar, Hungary | NGBAB033079  PAz77105SID0 | AL | W | SP |  |  |
| TSP57 | MVGB141 | W | HU | HUN-REN ATK MGI, Martonvasar, Hungary | NGBAB033080  PAz77106SID0 | A | R | SP |  |  |
| TSP58 | MVGB142 | W | HU | HUN-REN ATK MGI, Martonvasar, Hungary | NGBAB033081  PAz77107SID0 | AL | G | SP | **X** |  |
| TSP59 | MVGB143 | W | HU | HUN-REN ATK MGI, Martonvasar, Hungary | NGBAB033082  PAz77108SID0 | A | R | SP |  |  |
| TSP60 | MVGB144 | W | HU | HUN-REN ATK MGI, Martonvasar, Hungary | NGBAB033083  PAz77109SID0 | AL | W | SP |  |  |
| TSP61 | MVGB145 | W | HU | HUN-REN ATK MGI, Martonvasar, Hungary | NGBAB033084  PAz77110SID0 | A | R | SP | **X** |  |
| TSP62 | MVGB146 | F | HU | HUN-REN ATK MGI, Martonvasar, Hungary | NGBAB033085  PAz77111SID0 | A | W | SP |  |  |
| TSP63 | MVGB308 | F | HU | Plant Breeding and Acclimatization Institute, Radzikow, Poland | NGBAB033163  PAz77112SID0 | AL | W | SP |  |  |
| TSP64 | MVGB318 | F | HU | University of Hohenheim, Stuttgart, Germany | NGBAB033173  PAz77113SID0 | AL | R | SP |  |  |
| TSP65 | MVGB319 | W | HU | University of Hohenheim, Stuttgart, Germany | NGBAB033174  PAz77114SID0 | A | R | SP |  |  |
| TSP66 | MVGB353 | F | HU | HUN-REN ATK MGI, Martonvasar, Hungary | NGBAB033208  PAz77116SID0 | AL | R | SP | **X** |  |
| TSP67 | MVGB523 | F | HU | HUN-REN ATK MGI, Martonvasar, Hungary | NGBAB033374  PAz77118SID0 | AL | W | SP |  |  |
| TSP68 | MVGB524 | S | HU | HUN-REN ATK MGI, Martonvasar, Hungary | NGBAB033375  PAz77119SID0 | A | B | SP |  |  |
| TSP69 | MVGB525 | S | HU | HUN-REN ATK MGI, Martonvasar, Hungary | NGBAB033376  PAz77120SID0 | A | B | SP |  |  |
| TSP70 | MVGB526 | F | HU | HUN-REN ATK MGI, Martonvasar, Hungary | NGBAB033377  PAz77121SID0 | A | W | SP |  |  |
| TSP71 | MVGB527 | F | HU | HUN-REN ATK MGI, Martonvasar, Hungary | NGBAB033378  PAz77122SID0 | AL | R | SP | **X** |  |
| TSP72 | MVGB528 | F | HU | HUN-REN ATK MGI, Martonvasar, Hungary | NGBAB033379  PAz77123SID0 | AL | W | SP | **X** |  |
| TSP73 | MVGB529 | F | HU | HUN-REN ATK MGI, Martonvasar, Hungary | NGBAB033380  PAz77124SID0 | A | W | SP | **X** |  |
| TSP74 | WEIHENSTEPHAN | S | HU | Bundesanstalt für Züchtungsforschung an Kulturpflanzen (BAZ) Genbank, Braunschweig, Germany (in 2002 it was integrated into Institut für Pflanzengenetik und Kulturpflanzenforschung (IPK) Gatersleben) | NGBAB033407  PAz77125SID0 | AL | W | SP |  |  |
| TSP75 | MVGB557 | F | HU | Bundesanstalt für Züchtungsforschung an Kulturpflanzen (BAZ) Genbank, Braunschweig, Germany (in 2002 it was integrated into Institut für Pflanzengenetik und Kulturpflanzenforschung (IPK) Gatersleben) | NGBAB033408  PAz77126SID0 | AL | W | SP |  |  |
| TSP76 | MVGB855 | F | HU | Bundesanstalt für Züchtungsforschung an Kulturpflanzen (BAZ) Genbank, Braunschweig, Germany (in 2002 it was integrated into Institut für Pflanzengenetik und Kulturpflanzenforschung (IPK) Gatersleben) | NGBAB033695  PAz77127SID0 | A | G | SP |  |  |
| TSP77 | BLACK-BEARDED | W | AU | Australian Grains Genebank, Victoria, Australia | PAz82632SID0 | A | B | SP |  |  |
| TSP78 | WHITE BEARDLESS | W | AU | Australian Grains Genebank, Victoria, Australia | PAz82633SID0 | AL | W | SP | **X** |  |
| TSP79 | ALBISPICATUM | W | AU | Australian Grains Genebank, Victoria, Australia | PAz82635SID0 | A | W | SP |  |  |
| TSP80 | COERULEUM | W | AU | Australian Grains Genebank, Victoria, Australia | PAz82636SID0 | A | B | SP |  |  |
| TSP81 | ALBUM | W | AU | Australian Grains Genebank, Victoria, Australia | PAz82637SID0 | AL | W | SP |  |  |
| TSP82 | ALEFELDII | W | AU | Australian Grains Genebank, Victoria, Australia | PAz82638SID0 | AL | B | SP |  |  |
| TSP83 | VAVILOVII | W | AU | Australian Grains Genebank, Victoria, Australia | PAz82639SID0 | A | W | W |  |  |
| TSP84 | DUHAMELIANUM | W | AU | Australian Grains Genebank, Victoria, Australia | PAz82640SID0 | AL | R | SP |  |  |
| TSP85 | VULPINUM | W | AU | Australian Grains Genebank, Victoria, Australia | PAz82641SID0 | A | R | SP | **X** |  |
| TSP86 | ALBI-SPICATUM | W | AU | Australian Grains Genebank, Victoria, Australia | PAz82642SID0 | AL | W | SP |  |  |
| TSP87 | ALBO VELUTINUM | W | AU | Australian Grains Genebank, Victoria, Australia | PAz82643SID0 | A | W | SP |  |  |
| TSP88 | VULPINUM ALEF. | W | AU | Australian Grains Genebank, Victoria, Australia | PAz82644SID0 | A | R | SP |  |  |
| TSP89 | DUHAMELIANUM-MAZZ | W | AU | Australian Grains Genebank, Victoria, Australia | PAz82645SID0 | AL | G | SP | **X** |  |
| TSP90 | SAHARENSE | W | AU | Australian Grains Genebank, Victoria, Australia | PAz82646SID0 | AL | G | SP |  |  |
| TSP91 | ZOLLERNSPELZ | W | DE | University of Hohenheim, Stuttgart, Germany | PAz107295SID0 | AL | R | SP |  |  |
| TSP92 | BADENKRONE | W | DE | University of Hohenheim, Stuttgart, Germany | PAz107296SID0 | AL | R | SP |  |  |
| TSP94 | EBNERS-ROTKORN | W | DE | University of Hohenheim, Stuttgart, Germany | PAz107962SID0 | AL | R | SP |  |  |
| TSP95 | EMILIANO | W | DE | Cultivari Getreidezüchtungsforschung Darzau GmbH, Neu Darchau, Germany | PAz109308SID0 | AL | RB | SP |  |  |
| TSP96 | ALBERTINO | W | DE | Dr. Alter Pflanzenzucht und Versuchswesen, Felsberg, Germany | PAz112135SID0 | AL | R | SP |  |  |
| TSP98 | ROTTHWEILER-FRUEHKORN | W | SK | Research Institute of Plant Production, Piestany, Slovakia | PAz111372SID0 | AL | R | SP | **X** |  |
| TSP100 | ROTER-KOLBENDINKEL | W | SK | Research Institute of Plant Production, Piestany, Slovakia | PAz111375SID0 | AL | R | SP | **X** |  |
| TSP101 | BURGDORF 1 | W | SK | Research Institute of Plant Production, Piestany, Slovakia | PAz111376SID0 | AL | W | SP | **X** |  |
| TSP102 | RUBIOTA | W | SK | Research Institute of Plant Production, Piestany, Slovakia | PAz111377SID0 | AL | R | SP | **X** |  |
| TSP103 | CERALIO | W | SK | Research Institute of Plant Production, Piestany, Slovakia | PAz111378SID0 | AL | W | SP | **X** |  |
| TSP104 | STAKKET | W | SK | Research Institute of Plant Production, Piestany, Slovakia | PAz111379SID0 | A | R | SP |  |  |
| TSP105 | BRUN | W | SK | Research Institute of Plant Production, Piestany, Slovakia | PAz111380SID0 | AL | R | SP | **X** |  |
| TSP106 | SPELT-FRA-OLAND | W | SK |  |  | AL | R | W |  |  |
| TSP107 | PN-MISLINA | W | SK | Research Institute of Plant Production, Piestany, Slovakia | PAz111382SID0 | AL | R | SP | **X** |  |
| TSP108 | SPALDA | S | SK | Research Institute of Plant Production, Piestany, Slovakia | PAz111383SID0 | AL | W | W |  |  |
| TSP109 | CDC BAVARIA | S | SK | Research Institute of Plant Production, Piestany, Slovakia | PAz111384SID0 | A | B | SP |  |  |
| TSP110 | ZUGER DINKEL | S | SK | Research Institute of Plant Production, Piestany, Slovakia | PAz111385SID0 | AL | W | SP |  |  |
| TSP111 | SCHWARZER BEHAARTER WINTERKOLBEN-DINKELANS NORDLINGEN | W | HU | National Biodiversity and Gene Conservation Center, Tapioszele, Hungary | PAz111386SID0  RCAT002731 | AL | RB | SP |  |  |
| TSP112 | JENTIB | W | HU | National Biodiversity and Gene Conservation Center, Tapioszele, Hungary | PAz111387SID0  RCAT002732 | AL | W | SP |  |  |
| TSP113 | ROTTER-SOMMERKOLBEN | S | HU | National Biodiversity and Gene Conservation Center, Tapioszele, Hungary | PAz111388SID0  RCAT002739 | AL | R | SP | **X** |  |
| TSP114 | ARDENNE | W | BE | National Biodiversity and Gene Conservation Center, Tapioszele, Hungary | PAz81709SID0  RCAT002743 | AL | W | SP |  |  |
| TSP115 | RENVAL | W | HU | National Biodiversity and Gene Conservation Center, Tapioszele, Hungary | PAz111389SID0  RCAT002745 | AL | R | SP | **X** |  |
| TSP116 | ALTGOLD | W | CH | National Biodiversity and Gene Conservation Center, Tapioszele, Hungary | PAz81708SID0  RCAT002746 | AL | R | SP |  |  |
| TSP117 | LENZBURG | W | HU | National Biodiversity and Gene Conservation Center, Tapioszele, Hungary | PAz111390SID0  RCAT002750 | AL | W | SP |  |  |
| TSP118 | ROTER-GRANNENSPELZ | W | HU | National Biodiversity and Gene Conservation Center, Tapioszele, Hungary | PAz111391SID0  RCAT002752 | A | R | SP | **X** |  |
| TSP119 | WEISSER WINTER-GRANNENDINKEL ANS HOHENHEIM | W | AT | National Biodiversity and Gene Conservation Center, Tapioszele, Hungary | PAz111392SID0  RCAT003587 | A | W | SP |  |  |
| TSP120 | ALBIN | W | HU | National Biodiversity and Gene Conservation Center, Tapioszele, Hungary | PAz111393SID0  RCAT006081 | AL | W | SP |  |  |
| TSP121 | BLAUER-SAMTIGER-WINTERSPELZ | W | HU | National Biodiversity and Gene Conservation Center, Tapioszele, Hungary | PAz111394SID0  RCAT054345 | A | B | SP | **X** |  |
| TSP122 | BRAUNER-WINTER-GRANNENDINKEL-AUS-NOERDLINGEN | W | HU | National Biodiversity and Gene Conservation Center, Tapioszele, Hungary | PAz111395SID0  RCAT055693 | A | R | SP | **X** |  |
| TSP123 | KIPPERHAUS-WEISSER-SPELZ | W | BG | National Biodiversity and Gene Conservation Center, Tapioszele, Hungary | PAz111396SID0  RCAT055694 | AL | W | SP | **X** |  |
| TSP124 | BRAUNER- BEHAARTER-WINTER-GRANNENDINKEL | W | HU | National Biodiversity and Gene Conservation Center, Tapioszele, Hungary | PAz111397SID0  RCAT055918 | A | G | SP |  |  |
| TSP125 | LOHNAUER-SOMMERSPELZ | S | HU | National Biodiversity and Gene Conservation Center, Tapioszele, Hungary | PAz111398SID0  RCAT059286 | AL | R | SP |  |  |
| TSP126 | ROTER-KOLBENSPELZ | W | HU | National Biodiversity and Gene Conservation Center, Tapioszele, Hungary | PAz111399SID0  RCAT070256 | AL | R | SP |  |  |
| TSP127 | WAGGERSHAUSER HOHENHEIMER WEISSER KOLBEN | W | DE | IPK-Gatersleben, Genebank Department, Gatersleben, Germany | PAz111408SID0 | AL | W | SP |  |  |
| TSP128 | FUGGERS-BABENHAUSER-ZUCHTVEESEN | W | DE | IPK-Gatersleben, Genebank Department, Gatersleben, Germany | PAz111409SID0 | AL | R | SP | **X** |  |
| TSP129 | SPELZ-AUS-TZARIBORD | S | RU | IPK-Gatersleben, Genebank Department, Gatersleben, Germany | PAz111410SID0 | AL | W | SP |  |  |
| TSP130 | KIPPERHAUS-ROTER-SPELZ | W | DE | IPK-Gatersleben, Genebank Department, Gatersleben, Germany | PAz111412SID0 | AL | R | SP |  |  |
| TSP131 | STEINERS-ROTER-TIROLER-DINKEL | W | DE | IPK-Gatersleben, Genebank Department, Gatersleben, Germany | PAz111413SID0 | AL | RB | SP | **X** |  |
| TSP132 | TIROLER-ROTER-DINKEL-LOCKER | W | AT | IPK-Gatersleben, Genebank Department, Gatersleben, Germany | PAz111414SID0 | AL | R | SP |  |  |
| TSP133 | TIROLER-ROTER-DINKEL-DICHT | W | AT | IPK-Gatersleben, Genebank Department, Gatersleben, Germany | PAz111415SID0 | AL | R | SP |  |  |
| TSP134 | ZEINERS-SCHLEGELDINKEL | W | DE | IPK-Gatersleben, Genebank Department, Gatersleben, Germany | PAz111416SID0 | AL | W | SP |  |  |
| TSP135 | SCHLEGELDINKEL-WEISS | W | DE | IPK-Gatersleben, Genebank Department, Gatersleben, Germany | PAz111417SID0 | AL | W | SP |  |  |
| TSP136 | SCHWARZER BEHAARTER WINTER-GRANNENDINKEL | W | DE | IPK-Gatersleben, Genebank Department, Gatersleben, Germany | PAz111418SID0 | A | B | SP | **X** |  |
| TSP137 | RED WINTER SPELT | W | AU | IPK-Gatersleben, Genebank Department, Gatersleben, Germany | PAz111419SID0 | AL | R | SP |  |  |
| TSP138 | ROEHBERGS-FRUEHER-DINKEL | W | DE | IPK-Gatersleben, Genebank Department, Gatersleben, Germany | PAz111422SID0 | AL | R | SP |  |  |
| TSP139 | BAETTIG-NIEDERWILL | W | CH | IPK-Gatersleben, Genebank Department, Gatersleben, Germany | PAz111423SID0 | AL | R | SP | **X** |  |
| TSP140 | BRAUNER-SPELZ-AUS-SCHEFFLENZ | W | CH | IPK-Gatersleben, Genebank Department, Gatersleben, Germany | PAz111424SID0 | AL | R | SP |  |  |
| TSP141 | BURGHOF | W | DE | IPK-Gatersleben, Genebank Department, Gatersleben, Germany | PAz111425SID0 | AL | W | SP |  |  |
| TSP142 | FARNSBURG 6 | W | CH | IPK-Gatersleben, Genebank Department, Gatersleben, Germany | PAz111426SID0 | AL | R | SP |  |  |
| TSP143 | HUESLERS-NIEDERWILL-19 | W | CH | IPK-Gatersleben, Genebank Department, Gatersleben, Germany | PAz111427SID0 | AL | R | SP | **X** |  |
| TSP144 | LIESTAL-11 | W | CH | IPK-Gatersleben, Genebank Department, Gatersleben, Germany | PAz111428SID0 | AL | R | SP |  |  |
| TSP145 | STRICKHOF | W | DE | IPK-Gatersleben, Genebank Department, Gatersleben, Germany | PAz111429SID0 | AL | R | SP |  |  |
| TSP146 | RECHBERGS- BRAUNER-WINTERSPELZ | W | DE | IPK-Gatersleben, Genebank Department, Gatersleben, Germany | PAz111430SID0 | AL | R | SP |  |  |
| TSP147 | ROTTWEILER-DINKEL-ST1 | W | DE | IPK-Gatersleben, Genebank Department, Gatersleben, Germany | PAz111431SID0 | AL | RB | SP | **X** |  |
| TSP148 | ROTTWEILER-DINKEL-ST6 | W | DE | IPK-Gatersleben, Genebank Department, Gatersleben, Germany | PAz111432SID0 | AL | R | SP |  |  |
| TSP149 | VOEGELERS DINKEL WEISS | W | DE | IPK-Gatersleben, Genebank Department, Gatersleben, Germany | PAz111433SID0 | AL | W | SP |  |  |
| TSP150 | AUS KREUZUNG | S | DE | IPK-Gatersleben, Genebank Department, Gatersleben, Germany | PAz111435SID0 | AL | W | SP |  |  |
| TSP152 | STEINERS-ROTER-TIROLER | W | DE | IPK-Gatersleben, Genebank Department, Gatersleben, Germany | PAz111437SID0 | AL | RB | SP |  |  |
| TSP153 | ROTHENBURG 10 | W | DE | IPK-Gatersleben, Genebank Department, Gatersleben, Germany | PAz111438SID0 | AL | R | SP |  |  |
| TSP154 | COLL BBA 1305 | W | ES | IPK-Gatersleben, Genebank Department, Gatersleben, Germany | PAz111439SID0 | A | W | SP |  |  |
| TSP155 | ZEINERS-WEISSER-SCHLEGELDINKEL | W | DE | IPK-Gatersleben, Genebank Department, Gatersleben, Germany | PAz111440SID0 | AL | W | SP |  |  |
| TSP156 | BREGENZER ROTER SPELZ | W | DE | IPK-Gatersleben, Genebank Department, Gatersleben, Germany | PAz111441SID0 | AL | R | SP | **X** |  |
| TSP157 | POMMERS-HOHENAUER-KOLBENSPELZ | W | DE | IPK-Gatersleben, Genebank Department, Gatersleben, Germany | PAz111442SID0 | AL | R | SP |  |  |
| TSP158 | ELSENEGGER-WEISSKORN | W | CH | IPK-Gatersleben, Genebank Department, Gatersleben, Germany | PAz111443SID0 | AL | W | SP |  |  |
| TSP159 | MACHA VI | W | RU | IPK-Gatersleben, Genebank Department, Gatersleben, Germany | PAz111444SID0 | A | W | SP |  |  |
| TSP160 | SCHWABENSPELZ | W | DE | IPK-Gatersleben, Genebank Department, Gatersleben, Germany | PAz111445SID0 | AL | R | SP |  |  |
| TSP161 | TOESS 1B | W | CH | WBF-Agroscope, Switzerland | PAz111447SID0 | AL | R | SP | **X** |  |
| TSP162 | MUELLERS-GALBERGER | W | CH | WBF-Agroscope, Switzerland | PAz111448SID0 | AL | R | SP |  |  |
| TSP163 | WAGGERHAUSER WEISSEN KOLBEN | W | CH | WBF-Agroscope, Switzerland | PAz111449SID0 | AL | W | SP |  |  |
| TSP164 | WILLISAUER WEISSKORN | W | CH | WBF-Agroscope, Switzerland | PAz111454SID0 | AL | W | SP |  |  |
| TSP165 | ZEINERS-WEISSER SCHLEGELDINKEL-CH | W | CH | WBF-Agroscope, Switzerland | PAz111464SID0 | AL | W | SP | **X** |  |
| TSP166 | DEINISCHWANG | W | CH | WBF-Agroscope, Switzerland | PAz111465SID0 | AL | W | SP |  |  |
| TSP167 | ALSTROUM | W | CH | WBF-Agroscope, Switzerland | PAz111467SID0 | AL | W | SP |  |  |
| TSP168 | RECHBERGS-FRUEHEN-DINKEL | W | CH | WBF-Agroscope, Switzerland | PAz111468SID0 | AL | R | SP | **X** |  |
| TSP169 | HUESLER-ROTKRN | W | CH | WBF-Agroscope, Switzerland | PAz111470SID0 | AL | R | SP |  |  |
| TSP170 | BABENHAUSER ROTVESEN | W | CH | WBF-Agroscope, Switzerland | PAz111473SID0 | AL | R | SP |  |  |
| TSP171 | ROTER-SCHLEGEL DINKEL | W | CH | WBF-Agroscope, Switzerland | PAz111474SID0 | AL | R | SP |  |  |
| TSP172 | MURI-ROTKORN | W | CH | WBF-Agroscope, Switzerland | PAz81749SID0 | AL | R | SP |  |  |
| TSP173 | STRICKHOF ROTKORN | W | CH | WBF-Agroscope, Switzerland | PAz111476SID0 | AL | R | SP |  |  |
| TSP174 | STEINERS-ROTER-TIROLER-DINKEL-CH | W | CH | WBF-Agroscope, Switzerland | PAz111477SID0 | AL | RB | SP |  |  |
| TSP175 | GAIBERG-GELBER-BADISCHER-SPELZ | W | CH | WBF-Agroscope, Switzerland | PAz111478SID0 | AL | R | SP |  |  |
| TSP176 | LONIGO | W | CH | WBF-Agroscope, Switzerland | PAz111479SID0 | AL | R | SP |  |  |
| TSP177 | STOLZS BRAUNER WINTERKOLBEN SPELZ | W | CH | WBF-Agroscope, Switzerland | PAz111480SID0 | AL | RB | SP |  |  |
| TSP178 | SOFIA 1 | W | CH | WBF-Agroscope, Switzerland | PAz111482SID0 | A | W | SP |  |  |
| TSP179 | SAMMETIGER WINTER KOLBENSPELZ | W | CH | WBF-Agroscope, Switzerland | PAz111483SID0 | A | W | SP |  |  |
| TSP180 | ROTER-KOLBEN | W | CH | WBF-Agroscope, Switzerland | PAz111486SID0 | AL | R | SP | **X** |  |
| TSP181 | WARTENSEE | W | BE | WBF-Agroscope, Switzerland | PAz111488SID0 | AL | W | SP | **X** |  |
| TSP182 | SALEZ | W | CH | WBF-Agroscope, Switzerland | PAz111489SID0 | AL | R | SP | **X** |  |
| TSP183 | TORA | W | CH | WBF-Agroscope, Switzerland | PAz111490SID0 | AL | W | SP |  |  |
| TSP185 | RUEFENEN | W | CH | WBF-Agroscope, Switzerland | PAz111492SID0 | AL | W | SP | **X** |  |
| TSP186 | STEINERS-ROTER-TIROLER-CH | W | CH | WBF-Agroscope, Switzerland | PAz111493SID0 | AL | RB | SP |  |  |
| TSP187 | GUMPENSTEIN-BINKELWEIZEN | W | CH | WBF-Agroscope, Switzerland | PAz111494SID0 | A | G | SP |  |  |
| TSP188 | GUMPENSTEIN-GRANNENSPITZEN | W | CH | WBF-Agroscope, Switzerland | PAz111495SID0 | AL | RB | SP |  |  |

Where: Growing habit is W- winter, F- facultative or S- spring; Type of spike is A- awn or AL- awnless; Colour of spike is W- white, R-red, B- brown or G-grey; Form of spike is BW- bread wheat type, SP- spelt type or WH- wild hexaploid; Origin is AR- Argentina, AT- Austria, AU- Australia, BE- Belgium, BG- Bulgaria, CA- Canada, CH- Switzerland, CN- China, CS- Serbia-Montenegro, CZ- Czech Republic, DE- Germany, ES- Spain, FR- France, GB- United Kingdom, HU- Hungary, IT- Italy, KR- Korea, KZ- Kazahstan, PK- Pakistan, PL- Poland, RO- Romania, RU- Russia, SK- Slovakia, TR- Turkey, UA- Ukrajna or US- United States.
