# Supplementary material for: Genome-wide association analysis of fiber-related traits and identification of diagnostic SNP markers differentiating spelt wheat (Triticum spelta L.) from bread wheat
Source: Front Plant Sci. 2026 Aug 7;17:1907921. doi: 10.3389/fpls.2026.1907921 (PMC13493604; doi:10.3389/fpls.2026.1907921)
Supplement: Supplementary file 3 [file Table2.docx]

Supplementary Table S2. Mean, standard deviation, minimum and maximum values of thousand kernel weight and fiber components (total (TOT) and water extractable (WE) pentosan, β-glucan, fructan) in selected spelt (n=50) and bread wheat (n=50) genotypes harvested in 2021 and 2022.

| **Specie** |  |  |  | **TGW**  **(g/1000 kernel)** | **Total pentosan**  **(mg/g)** | **WE pentosan**  **(mg/g)** | **β glucan content**  **(mg/g)** | **Fructan content**  **(%)** |
| --- | --- | --- | --- | --- | --- | --- | --- | --- |
| Spelt | 2021 | min-max | n=50 | 25.78-54.71 | 11.87-20.63 | 5.66-11.45 | 4.19-6.36 | 0.39-1.51 |
|  |  | Mean |  | 38.86 | 15.45 | 7.75 | 5.42 | 0.96 |
|  |  | SD |  | 6.31 | 1.70 | 1.40 | 0.53 | 0.22 |
|  | 2022 | min-max | n=50 | 32.81-50.90 | 11.42-19.82 | 3.39-9.63 | 3.13-7.23 | 0.48-1.17 |
|  |  | Mean |  | 40.89 | 15.30 | 6.44 | 5.54 | 0.84 |
|  |  | SD |  | 4.20 | 1.90 | 1.48 | 0.81 | 0.17 |
|  | all | Mean | n=100 | 39.87 | 15.38 | 7.10 | 5.48 | 0.90 |
|  |  | SD |  | 5.43 | 1.80 | 1.58 | 0.68 | 0.20 |
|  | **2021, 2022** | **LSD** |  | **1.50^n.s.^** | **0.50 ^n.s.^** | **0.69***** | **0.19^n.s.^** | **0.08**** |
| Bread Wheat | 2021 | min-max | n=50 | 29.34-52.62 | 10.46-20.85 | 4.48-13.61 | 4.77-10.15 | 0.42-1.52 |
|  |  | Mean |  | 41.89 | 14.65 | 8.35 | 6.61 | 0.99 |
|  |  | SD |  | 5.73 | 2.47 | 1.95 | 1.14 | 0.25 |
|  | 2022 | min-max | n=50 | 27.55-51.27 | 11.22-19.83 | 3.78-11.4 | 4.34-9.35 | 0.56-1.53 |
|  |  | Mean |  | 40.02 | 15.28 | 7.49 | 6.98 | 0.98 |
|  |  | SD |  | 5.48 | 2.43 | 1.90 | 1.02 | 0.21 |
|  | all | Mean | n=100 | 40.95 | 14.96 | 7.92 | 6.80 | 0.98 |
|  |  | SD |  | 4.56 | 1.43 | 1.09 | 0.40 | 0.41 |
|  | **2021, 2022** | **LSD** |  | **1.56 ^n.s.^** | **0.68^n.s.^** | **0.54*** | **0.30^n.s.^** | **0.06^n.s.^** |
|  | all | **LSD** |  | **1.46^n.s.^** | **0.52 ^n.s.^** | **0.62 **** | **0.37***** | **0.06*** |
|  | 2021 | **LSD** |  | **1.68*** | **0.59^n.s.^** | **0.47^n.s.^** | **0.43***** | **0.07^n.s.^** |
|  | 2022 | **LSD** |  | **1.36^n.s.^** | **0.61^n.s.^** | **0.70**** | **0.44***** | **0.09***** |

*, **, *** significant at 0.05, 0.01 and 0.001 probability level, respectively, n.s.- not significant, LSD- Least Significant Difference
